# New Iridoid Glucosides from *Caryopteris incana* (Thunb.) Miq. and Their α-Glucosidase Inhibitory Activities

**DOI:** 10.3390/molecules21121749

**Published:** 2016-12-21

**Authors:** Xu-Dong Mao, Gui-Xin Chou, Sen-Miao Zhao, Cheng-Gang Zhang

**Affiliations:** 1 The MOE Key Laboratory of Standardization of Chinese Medicines, and SATCM Key Laboratory of New Resources and Quality Evaluation of Chinese Medicines, Institute of Chinese Materia Medica, Shanghai University of Traditional Chinese Medicine, Shanghai 201210, China; maoxd1993@163.com (X.-D.M.); zhaoziyang1989@163.com (S.-M.Z.); chenggangzhang88@163.com (C.-G.Z.); 2 Shanghai R&D Center for Standardization of Chinese Medicines, Shanghai 201203, China

**Keywords:** *Caryopteris incana*, iridoid glucosides, type-II diabetes mellitus, α-glucosidase inhibition

## Abstract

In our continued investigations of the plant *Caryopteris incana*, five new iridoid glucosides **1**–**5**, including two *cis-trans*-isomers, **3** and **4**, along with six known compounds **6**–**11**, were isolated from the *n*-butyl alcohol (*n*-BuOH) soluble fraction of whole dried material of *Caryopteris incana*. Their structures were established by a combination of spectroscopic techniques, including 1D and 2D NMR and high resolution electrospray ionization mass spectroscopy (HR-ESI-MS). Furthermore, all isolates were evaluated for their yeast α-glucosidase inhibitory effects. Among these compounds, **4**–**8** and **10** exhibited potent inhibition of α-glucosidase.

## 1. Introduction

The incidence of obesity and diabetes in humans is high and continues to increase. In particular, type-II diabetes mellitus (T2DM) is one of the most common chronic diseases in nearly all countries, and it continues to be an increasing international health burden and hidden killer [1]. Since the early 1990s, comparatively effective glucosidase inhibitors have been generally available to type-II diabetics in order to depress postprandial hyperglycemia induced by the digestion of carbohydrates in the intestines [2]; however, they are accompanied by side effects.

Traditional Chinese medicines are known to be a potential rich source, and chemical pool, for finding effective treatment agents with mild or no side effects. *Caryopteris incana* (Thunb.) Miq., known in China as “Lanxiangcao”, is a Chinese folk medicine for the relief of colds, pertussis, rheumatic pains, bronchitis, eczema, gastroenteritis, itchy skin, and venomous snake bites [3]. Up to now, there have been many reports regarding its chemical constituents, such as diterpenes [4], iridoids [5], flavonoids [6], and phenolic glycosides [7].

Recently, we reported 12 new diterpenes caryopincaolide A–L, including four, new, rearranged abietanes, caryopincaolide A–D, with unprecedented skeletons, and twenty-eight known diterpenes, isolated from the petroleum ether and ethyl acetate soluble fractions of whole plant of *C. incana*; additionally, some compounds exhibited moderate inhibitory effects against dipeptidyl peptidase IV (DPP-IV) [8]. Furthermore, in the previous literature, some iridoid glycosides and phenylpropanoid glycosides displayed potential α-glucosidase inhibitory activities [9,10]. Thus, the aim of the present work was to continue phytochemical investigations on the *n*-butyl alcohol soluble fraction of this plant in order to discover effective hypoglycaemic agents to remedy serious T2DM.

**Figure 1:**
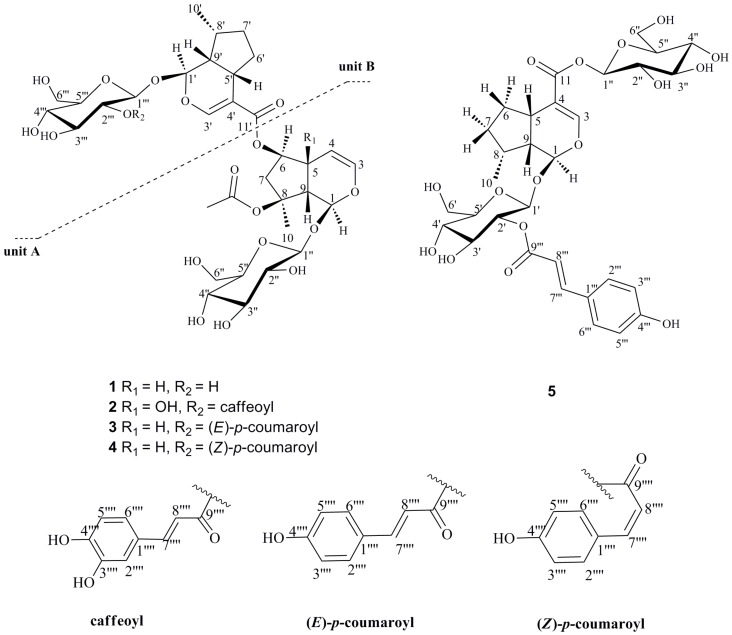
Chemical structures of compounds **1**–**5**.

In this paper, the isolation and structural identification of five new iridoid glycosides are described: Caryocanoside B (**1**), 5-hydroxy-2′′′-*O*-caffeoylcaryocanoside B (**2**), 2′′′-*O*-(*E*)-*p*-coumaroyl caryocanoside B (**3**), 2′′′-*O*-(*Z*)-*p*-coumaroyl caryocanoside B (**4**), and 2′-*O*-(*E*)-*p*-coumaroyl asystasioside A (**5**) (Figure 1). Six known compounds were isolated and determined by comparison of the physical data with those reported in the literature: Two iridoid glycosides, 8-*O*-acetylharpagide (**6**) [11] and 8-acetyl-6′-*O*-(*p*-coumaroyl) harpagide (**7**) [12], two phenethyl alcohol glycoside, galactosylmartynoside (**8**) [13] and 6‴-*O*-feruloylincanoside D (**9**) [14], an abietatriene-type diterpene glycoside, ajugaside A (**10**) [13], and a flavonoid luteolin 7-*O*-glucoside (**11**) [15], respectively. All compounds were tested for in vitro α-glucosidase inhibitory activity; compounds **4** and **10** possess α-glucosidase inhibitory effects with IC_50_ values of 0.377 mM and 0.328 mM, respectively.

## 2. Results

**Figure 2:**
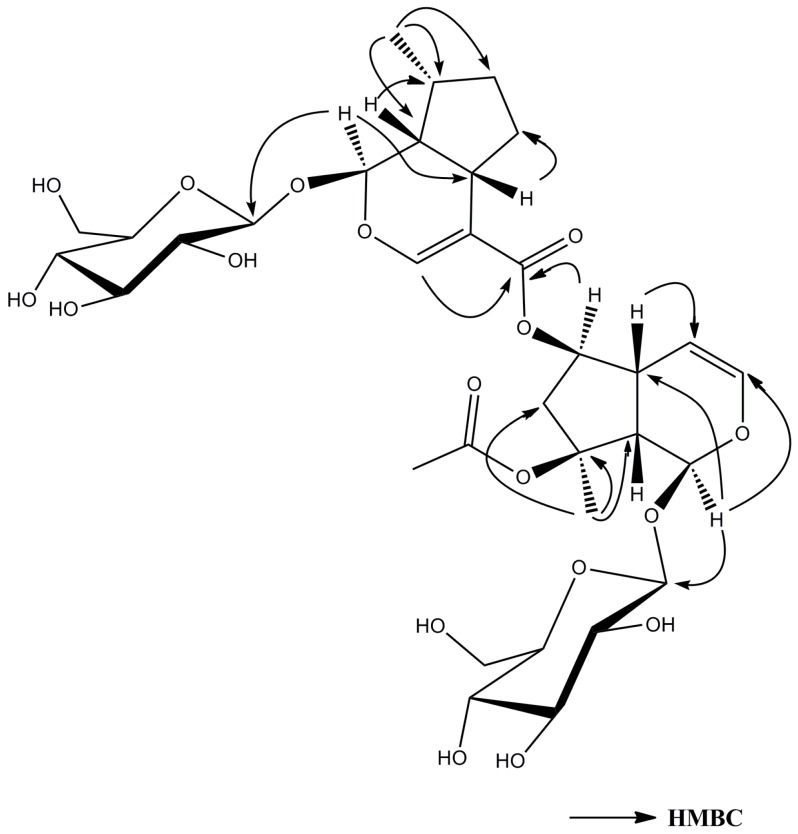
Selected heteronuclear multiple bond correlations (HMBC) for compound **1**.

**Figure 3:**
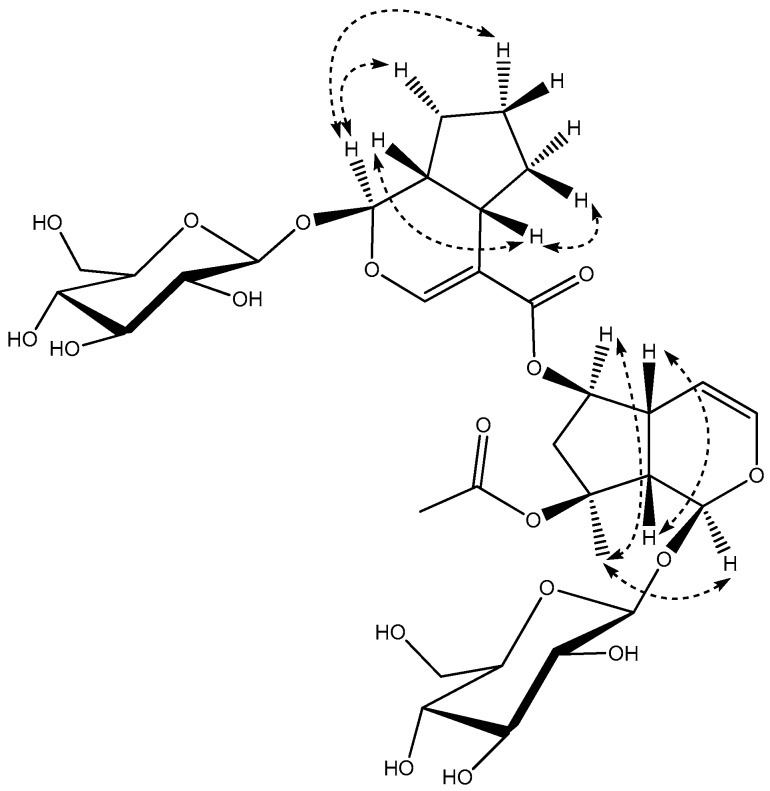
Selected Nuclear Overhauser Effect Spectroscopy (NOESY) correlations for compound **1**.

Compound **1**, was obtained as a yellow powder, ${\left[\alpha \right]}_{D}^{20}$ = −0.098 (*c* 0.1, MeOH), its molecular formula, C_33_H_48_O_18_, was established by its high resolution electrospray ionization mass spectroscopy (HR-ESI-MS) ion peak (*m/z* 731.2802 [M − H]^−^), which corresponded to 10 units of unsaturation. The absorptions in the infrared (IR) spectrum at 3357 and 1633 cm^−1^ showed the existence of hydroxy and carbonyl groups. The ^1^H NMR spectrum (Table 1) of CD_3_OD revealed characteristic of bis-iridoid glucoside signals attributed to the disubstituted olefinic protons at δ_H_ 6.26 (1H, dd, *J* = 2.4, 6.0 Hz, H-3), δ_H_ 4.77 (1H, brd, *J* = 6.0 Hz, H-4), a trisubstituted olefinic proton at δ_H_ 7.42 (1H, s, H-3′), two hemiacetal protons at δ_H_ 5.87 (1H, s, H-1), δ_H_ 5.47 (1H, d, *J* = 4.4, H-1′) together with two β-anomeric protons at δ_H_ 4.68 (1H, d, *J* = 8.0 Hz, H-1″), δ_H_ 4.64 (1H, d, *J* = 8.0 Hz, H-1‴). The ^13^C NMR spectrum (Table 1) showed the presence of two β-glucopyranosyl moieties and two carbonyl groups, suggesting that **1** was an ester dimmer of iridoid glycoside. By comparison of the ^13^C-NMR data with those known iridoid glycosides, one unit (A) (Figure 1) of **1** was determined to be ajugoside [16,17], and another (B) (Figure 1) to be 7-deoxy-8-*epi*-loganic acid [18]. The main difference between unit A and ajugoside was that the C-6 in unit A moved to the downfield region (from δ_C_ 76.9 to 79.4), and unit B, between 7-deoxyloganic acid, was an upfield shift of C-11′ in unit B (from δ_C_ 172.2 to 168.4), indicating that units A and B were connected via esterification between the hydroxyl group at C-6 of ajugoside (unit A) and the carboxylic acid group of 7-deoxyloganic acid (unit B), which was confirmed by heteronuclear multiple bond correlation (HMBC) (Figure 2) correlation between δ_H_ 4.97 (H-6) and δ_C_ 168.4 (C-11′). HMBC of **1** showed the following correlations: H-1/C-5, C-8; H-3/C-1, C-5; H-4/C-6, C-9; H-5/C-4, C-6, C-8; H-7/C-5, C-9, C-10; H-10/C-8 and H-1′/C-5′, C-8’; H-3′/C-1′, C-5′, C-11′; H-7′/C-5′, C-9′, C-10′; H-10′/C-8′, which further proves the existence of unit A and B. The Nuclear Overhauser Effect Spectroscopy (NOESY) (Figure 3) experiment established the stereochemistry of **1**; for unit A, the correlations between δ_H_ 5.87 (1H, s, H-1) and 1.58 (3H, s, H-10), δ_H_ 4.97 (1H, d, H-6) and H-10; and between δ_H_ 2.98 (1H, brd, H-5) with 2.81 (1H, brd, H-9) revealed that H-1, H-6, and H-10 were on the same face, an α-orientation, whereas H-5 and H-9 were on the opposite face, a β-position, showing that the relative configuration of unit A was identical to ajugoside. For unit B, obvious Nuclear Overhauser Effect (NOE) cross-peaks of δ_H_ 5.47 (1H, d, H-1′) with 1.08 (3H, d, H-10′), H-1′ with δ_H_ 1.38 (1H, m, Hα-7); and δ_H_ 2.91 (1H, brdd, H-5′) with 2.07 (1H, m, Hβ-6′), H-5′ with δ_H_ 2.27 (1H, m, H-9′) were observed, demonstrating H-5′and H-9′ to be of a β-orientation, and H-1′ and H-10′ to be in the α-position. The relative configuration of unit B was also the same as 7-deoxy-8-*epi*-loganic acid. Thus, compound **1** was an ester dimer connected by an ester bond and the bridging ester bond was located at the 6-position of ajugoside, and was named caryocanoside B (shown in Figure 1).

Compound **2**, a yellow powder, ${\left[\alpha \right]}_{D}^{20}$ = −0.266 (*c* 0.1, MeOH), with a molecular formula of C_42_H_54_O_22_, was established from HR-ESI-MS data ([M − H]^−^, *m/z* 909.3038), requiring 15 degrees of unsaturation. IR spectra indicated the presence a hydroxyl group at 3357 cm^−1^, a carbonyl group at 1704 cm^−1^ and an olefinic group at 1669 cm^−1^ for **2**. The NMR spectra of **2** (Table 1) had a close structural similarity to those of compound **1**, except for the addition of a hydroxyl at C-5 (δ_C_ 72.7), and a caffeoyl group approved by a set of ABX-type aromatic protons at δ_H_ 7.06 (1H, d, *J* = 2.0 Hz, H-2′′′′), 6.97 (1H, dd, *J* = 8.0, 2.0 Hz, H-6′′′′) and 6.79 (1H, d, *J* = 8.0 Hz, H-5′′′′); δ_C_ 168.3 (C-9′′′′), δ_C_ 114.9 (C-8′′′′), and δ_C_ 147.1 (C-7′′′′). The connectivity of these fragments was supported by a careful analysis of 2D-NMR (heteronuclear single quantum correlation (HSQC), ^1^H-^1^H correlation spectroscopy (^1^H-^1^H COSY), and HMBC) spectra, which corroborated that the caffeoyl group was located at C (2′′′), based on the HMBC correlation of δ_H_ 4.79 (1H, d, H-2′′′) with δ_C_ 168.3 (C-9′′′′). The relative configuration of compound **2** was established by its NOESY, and is the same as **1** (All spectroscopic data are available from Supplementary Material). Therefore, structure **2** was elucidated, named 5-hydroxy-2′′′-*O*-caffeoylcaryocanoside B, and is shown in Figure 1.

Compound **3** was isolated as a white amorphous powder, ${\left[\alpha \right]}_{D}^{20}$ = −0.168 (*c* 0.1, MeOH), and its molecular formula of C_42_H_54_O_20_ was determined from HR-ESI-MS data (*m/z* 877.3147 [M − H]^−^). The similarity of the IR and NMR data (Table 1) of **3** and **1**, suggested that **3** was also a derivative of **1**. The main difference was that **3** presented a *p*-coumaroyl group, with signals at δ_H_ 6.26 (1H, d, *J* = 15.6 Hz, H-8′′′′), δ_H_ 7.56 (1H, d, *J* = 15.6 Hz, H-7′′′′), δ_H_ 7.47 (2H, d, *J* = 8.4 Hz, H-2′′′′ and H-6′′′′), δ_H_ 6.83 (2H, d, *J* = 8.4 Hz, H-3″′ and H-5′′′′); δ_C_ 168.1 (C-9′′′′), 115.0 (C-8′′′′), 146.5 (C-7′′′′), 127.2 (C-1′′′′), 131.4 (C-2′′′′ and C-6′′′′), 116.9 (C-3′′′′ and C-5′′′′), and 161.2 (C-4′′′′). Additionally, the coumaroyl group was attached at C-2′′′, as confirmed by HMBC correlation of δ_H_ 4.79 (1H, t, H-2‴) with δ_C_ 168.1 (C-9′′′′). The relative configuration of **3** was identical to that of **1**, which was determined from its NOESY spectrum. Thus, structure **3** was elucidated as 2′′′-*O*-(*E*)-*p*-coumaroyl caryocanoside B and is shown in Figure 1.

Compound **4** was obtained as a white amorphous powder; ${\left[\alpha \right]}_{D}^{20}$ = −0.09 (*c* 0.1, MeOH). The HR-ESI-MS data (*m/z* 901.3092 [M + Na]^+^) and ^13^C NMR data (Table 1) of **4** indicated that **4** has the same molecular formula as that of compound **3**. The only significant difference was the replacement of the (*E*)-*p*-coumaroyl group in **3** by the (*Z*)-*p*-coumaroyl group in **4**. This was supported by the coupling constant (*J* = 12.8 Hz) of H-7′′′′ and H-8′′′′. Therefore, compound **4** was determined to be a *cis*-isomer of **3**, and was named as 2′′′-*O*-(*Z*)-*p*-coumaroyl caryocanoside B (shown in Figure 1).

**Figure 4:**
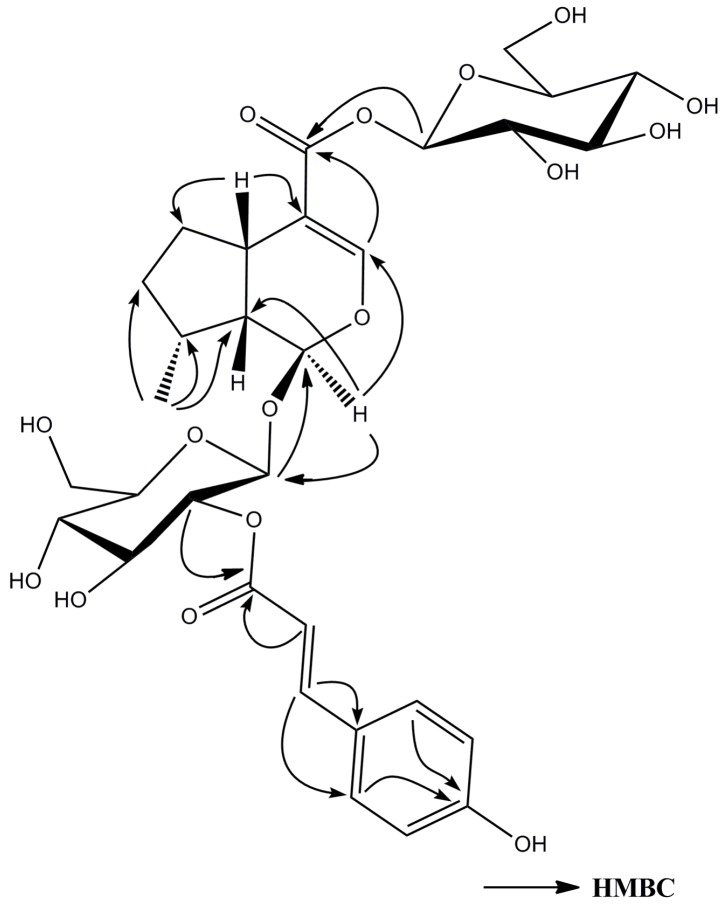
Selected HMBC correlations for compound **5**.

**Figure 5:**
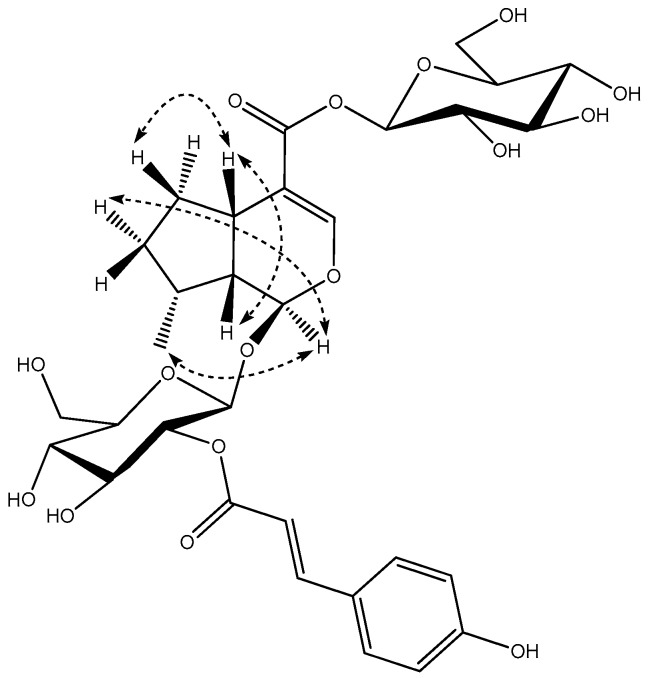
Selected NOESY correlations for compound **5**.

Compound **5** was obtained as a pale yellow powder; ${\left[\alpha \right]}_{D}^{20}$ = −0.058 (*c* 0.1, MeOH). It was shown to have a molecular formula of C_31_H_40_O_16_ from the [M − H]^−^ ion at *m/z* 667.2257, determined by HR-ESI-MS. The IR spectrum indicated the presence of a hydroxy at 3357 cm^−1^, carbonyl groups at 1698 cm^−1^, an olefinic group at 1632 cm^−1^, and a phenyl at 1603 cm^−1^. The similarity in the NMR spectroscopic data of **5** (Table 2) and those of asystasioside A [19] suggested that **5** is a derivative of asystasioside A. Interpretation of the NMR spectra of **5** allowed us to find an additional (*E*)-*p*-coumaroyl group in its structure; key signals at δ_H_ 6.29 (1H, d, *J* = 15.6 Hz, H-8′′′), δ_H_ 7.60 (1H, d, *J* = 15.6 Hz, H-7′′′), δ_H_ 7.52 (2H, d, *J* = 8.4 Hz, H-2′′′ and H-6′′′), and δ_H_ 6.83 (2H, d, *J* = 8.4 Hz, H-3′′′ and H-5′′′). The correlation between δ_H_ 4.81 (1H, overlap, H-2′) with δ_C_ 168.5 (C-9′′′) in the HMBC data (Figure 4) of **5** showed that the coumaroyl group was located at C-2′. In addition, the HMBC correlations from δ_H_ 4.83 (1H, overlap, H-1′) to δ_C_ 95.6 (C-1), and from δ_H_ 5.08 (1H, d, *J* = 8.4 Hz, H-1″) to δ_C_ 167 (C-11), supported that two β-glucopyranosyl moieties were at positions C-1 and C-11. The stereochemistry of **5** was determined from its NOESY spectrum (Figure 5), which displayed NOE cross-peaks of δ_H_ 5.50 (1H, d, H-1) with 1.24 (1H, m, Hα-7), H-1 with δ_H_ 1.03 (3H, d, H-10); δ_H_ 2.82 (1H, m, H-5) with 1.92 (1H, m, Hβ-6), H-5 with δ_H_ 2.27 (1H, m, H-9), indicated that **5** had the same relative configuration as asystasioside A. Thus, on the basis of the above-mentioned data, the structure of **5** was established as 2′-*O*-(*E*)-*p*-coumaroyl asystasioside A.

**Figure 6:**
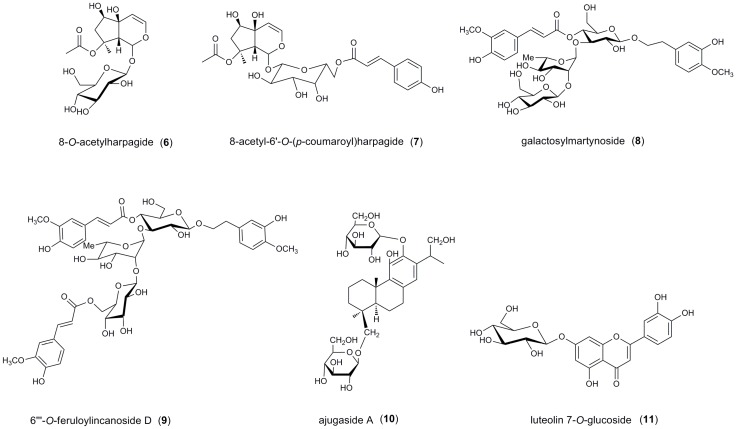
Chemical structures of isolated compounds **6**–**11**.

Compounds **6**–**11** (Figure 6) were identified by comparison with the literature. They were identified as 8-*O*-acetylharpagide (**6**), 8-acetyl-6′-*O*-(*p*-coumaroyl) harpagide (**7**), galactosylmartynoside (**8**), 6′′′-*O*-feruloylincanoside D (**9**), ajugaside A (**10**), and luteolin 7-*O*-glucoside (**11**).

The isolated compounds were evaluated for their inhibitory effects against α-glucosidase. Compounds **4** and **10** exhibited stronger inhibitory effects against α-glucosidase than the positive control, acarbose, with IC_50_ values of 0.377 mM and 0.328 mM, respectively (Table 3).

## 3. Experimental Section

### 3.1. General

IR spectra were measured on a PerkinElmer FT-IR spectrometer (PerkinElmer, Shanghai, China). Optical rotations were measured on an Autopol VI (Rudolph Research Analytical, Hackettstown, NJ, USA). A Bruker AV-400 spectrometer and Bruker AV-600 spectrometer (Bruker Co., Rheinstetten, Germany) were used for NMR spectroscopy, with tetramethylsilane (TMS) (Sigma-Aldrich Co., St. Louise, MO, USA) as the internal standard. HR-ESI-MS spectra were obtained on a Waters UPLC Premior QTOF spectrometer (Waters Co., Milford, MA, USA). Column chromatography (CC) was performed with silica gel (200–300 mesh; Qingdao Marine Chemical Factory, Qingdao, China), Sephadex LH-20 (GE Healthcare Bio-Sciences AB, Uppsala, Sweden), and YMC gel ODS-A-HG (50 μm, YMC Co., Ltd., Kyoto, Japan). Thin-layer chromatography (TLC) was conducted on silica gel GF254 plates (Qingdao Marine Chemical Factory, Qingdao, China). Preparative HPLC was performed on an Agilent 1260 series instrument (Agilent Co., Santa Clara, CA, USA) cooperated with a Shiseido Capcellpak Prep C18 ODS column (250 × 20 mm, 5 μm, Shiseido Co., Tokyo, Japan) with a flow rate of 16 mL∙min^−1^.

### 3.2. Plant Materials

Plant materials of *Caryopteris incana* (Thunb.) Miq. were harvested in Hexian County of Anhui Province, the People’s Republic of China, in September 2012. The fresh material was air-dried and ground to coarse powder. The identification of plant materials was verified by Prof. Qingshan Yang of Anhui University of Traditional Chinese Medicine. A voucher specimen (no. 20120915-1) was deposited in the Shanghai R&D Center for Standardization of Traditional Chinese Medicines, Shanghai, 201203, China.

### 3.3. Extraction and Isolation

The dried and crushed plants of *Caryopteris incana* (Thunb.) Miq. (19.5 kg) were completely extracted with EtOH at room temperature; the ethanolic extract was concentrated under reduced pressure, the residue was dissolved in hot water (10 L, 60 °C), and partitioned successively with petroleum ether (PE), ethyl acetate (EtOAc), and butyl alcohol (*n*-BuOH) in the same volume, 3–4 times in order to get the PE-(234 g), EtOAc-(355 g) and *n*-BuOH soluble (200 g) fractions. In this part, we performed continuous research on the *n*-BuOH soluble fraction of *C.* *incana*.

The *n*-BuOH soluble fraction (200 g) was subjected to column chromatography (CC) over silica gel (100–200 mesh), eluted with a gradient mixture of EtOAc/MeOH/H_2_O (15:2:1, 10:2:1, 8:2:1), to afford ten fractions 1–10. Fractions 2–5 were subjected to octadecyl silane (ODS) CC eluting with a gradient of MeOH/H_2_O (5:95–60:40, *v/v*) to give four sub-fractions (Fr. I–Fr. IV), Fr. I, Fr. II, and Fr. IV were further chromatographed on a Sephadex LH-20 column using MeOH/H_2_O (80:20, *v/v*) as eluent to afford compounds **6** (40 mg), 2 (3 mg) and 8 (15 mg), respectively; Fr. III was separated by preparative TLC (developer: EtOAc/MeOH/H_2_O 10:2:1; eluent: Acetone), followed by a Sephadex LH-20 column and eluted with (MeOH/H_2_O 80:20, *v/v*) to yield compound **1** (10 mg). Fr. 6–Fr. 8 were subjected to ODS CC eluting with a gradient of MeOH/H_2_O (5:95–50:50, *v/v*) to give three sub-fractions (Fr. V–Fr. VII); Fr. V was chromatographed on a Sephadex LH-20 column using MeOH/H_2_O (80:20, *v/v*) as eluent to acquire compound **11** (9 mg), followed by prep-HPLC on a Shiseido Capcellpak Prep C18 column, using acetonitrile/H_2_O (30:1, 16 mL∙min^−1^, detection at 254 nm) to afford compound **5** (5 mg); retention time was 15 min. Fr. VI was chromatographed on a Sephadex LH-20 column using MeOH/H_2_O (80:20, *v/v*) as eluent to get a mixture of compounds **3** and **4**, followed by prep-HPLC on a Shiseido Capcellpak Prep C18 column using MeOH/H_2_O (29:1, 16 mL∙min^−1^, detection at 254 nm) to afford compound **3** (11 mg) and compound **4** (4 mg); the retention time of compounds **3** and **4** were 11 and 13 min, respectively. Fr. VII was subjected to the same method as Fr. VI, and produced compounds **7** (6 mg), **9** (13 mg) and **10** (7 mg); the retention times of compounds **7**, **9** and **10** were 10, 16, and 13 min, respectively. (prep-HPLC detected at 254 nm, acetonitrile/H_2_O, 27:1).

### 3.4. Spectroscopic Data

*Caryocanoside B* (**1**) was obtained as a yellow amorphous powder; ${\left[\alpha \right]}_{D}^{20}$ = −0.098 (*c* 0.1, MeOH); IR ν_max_: 3357, 2921, 2851, 1704, 1633, 1422, 1369, 1274, 1194, 1071, 1010, 901, and 859 cm^−1^; HR-ESI-MS *m/z* 731.2802 [M − H]^−^, (calcd. for 731.2762); ^1^H- and ^13^C-NMR data, see Table 1.

*5-Hydroxy-2*′′′*-O-caffeoylcaryocanoside B* (**2**) was obtained as a yellow amorphous powder; ${\left[\alpha \right]}_{D}^{20}$ = –0.266 (*c* 0.1, MeOH); IR ν_max_: 3357, 1703, 1669, 1599, 1515, 1373, 1233, 1186, 1067, 1011, 901, 851, 810, and 773 cm^−1^; HR-ESI-MS *m/z* 909.3038 [M − H]^−^, (calcd. for 909.3040); ^1^H- and ^13^C-NMR data, see Table 1.

*2*′′′*-O-(E)-p-Coumaroyl caryocanoside B* (**3**) was obtained as a white amorphous powder; ${\left[\alpha \right]}_{D}^{20}$ = −0.168 (*c* 0.1, MeOH); IR ν_max_: 3313, 2949, 2162, 1979, 1702, 1646, 1603, 1587, 1515, 1444, 1365, 1326, 1271, 1171, 1009, 973, 897, and 836 cm^−1^; HR-ESI-MS *m/z* 877.3147 [M − H]^−^, (calcd. for 877.3130); ^1^H- and ^13^C-NMR data, see Table 1.

2′′′-*O*-(*Z*)-*p*-coumaroyl caryocanoside B (**4**) was obtained as a white amorphous powder; ${\left[\alpha \right]}_{D}^{20}$ = −0.09 (*c* 0.1, MeOH); IR ν_max_: 3358, 2921, 2851, 1704, 1633, 1604, 1514, 1424, 1370, 1261, 1190, 1067, 1008, 901, 854, 800, and 766 cm^−1^; HR-ESI-MS *m/z* 901.3092 [M + Na]^+^, (calcd. for 901.3106); ^1^H- and ^13^C-NMR data, see Table 1.

2′-*O*-(*E*)-*p*-coumaroyl asystasioside A (**5**) was obtained as a yellow amorphous powder; ${\left[\alpha \right]}_{D}^{20}$ = −0.058 (*c* 0.1, MeOH); IR ν_max_: 3357, 2922, 2851, 2161, 1698, 1632, 1603, 1514, 1425, 1263, 1169, 1061, 1024, 901, 834, 764 and 700 cm^−1^; HR-ESI-MS *m/z* 667.2257 [M − H]^−^, (calcd. for 667.2238); ^1^H- and ^13^C-NMR data, see Table 2.

### 3.5. Alpha-Glucosidase Inhibitory Assay

The α-glucosidase inhibition assay was performed according to a slightly modified, previously reported method [20]; α-Glucosidase (0.1 U/mL) was dissolved in potassium phosphate buffer (pH 6.8) as the enzyme solution. The tested compounds (50 μL, 5 mM) dissolved in potassium phosphate buffer containing 3% DMSO were mixed with 50 μL of enzyme solution. After incubation at 37 °C for 10 min, a *p*-nitrophenyl-α-glucopyranoside (*p*NPG) solution (100 μL) (5.0 mM *p*NPG in 0.1 M potassium phosphate buffer (pH 6.8)) was added. The enzymatic reaction proceeded at 37 °C for 20 min and was terminated by the addition of 100 μL of 0.2 M Na_2_CO_3_. 4-nitrophenol absorption was immediately measured at 405 nm by using a microplate reader. The experiments were performed in triplicate. The percent inhibition of α-glucosidase was calculated as inhibition rate (%) = 100 × [1 − (A_sample_ − A_s-blank_)/(A_control_ − A_blank_)]. The α-glucosidase from *Saccharomyces cerevisiae*, *p*NPG, and acarbose were purchased from Sigma-Aldrich.

## 4. Conclusions

Five new iridoid glucosides, **1**–**5**, including two (*cis/trans*) isomers, together with six known compounds were isolated from the whole plant of *C.* *incana*. The discovery of these compounds further expands our knowledge of the structural diversity of the glycosides produced by the plant. Furthermore, compounds **4**–**8** and **10** exhibited a significant *α*-glucosidase inhibitory activity, with IC_50_ values from 3.35 to 0.33 mM. 2′′′-*O*-(*Z*)-*p*-coumaroyl caryocanoside B (**4**) and ajugaside A (**10**) manifested the highest activities with IC_50_ values at 0.38 and 0.33 mM. Two isomers (**3** and **4**) showed distinct results to inhibition of α-glucosidase; only *cis*-isomer (**4**) had a potent effect. This mechanism needs to be further investigated. Combined with the research, which was completed by our predecessors, the potential hypoglycaemic activity of *C.* *incana* should be substantiated, and this plant could be regarded as a suitable herb-derived drug for the prevention and treatment of T2DM.

## Figures and Tables

**Table 1:** ^1^H- and ^13^C-NMR data for **1**–**4**.

Position	1 ^a^		2 ^a^		3 ^b^		4 ^a^	
	δ_H_ (*J*)	δ_C_	δ_H_ (*J*)	δ_C_	δ_H_ (*J*)	δ_C_	δ_H_ (*J*)	δ_C_
1	5.87 (s)	94.0	6.03 (s)	94.2	5.80 (s)	94.0	5.8 (s)	94.1
3	6.26 (dd, 2.4, 6.0)	142.2	6.35 (d, 6.4)	144.0	6.17 (d, 6.6)	141.9	6.25 (dd, 6.4, 2.4)	142.3
4	4.77 (brd, 6.0)	103.0	4.81 (brd, 8.0)	106.2	4.36 (d, 6.0)	103.0	4.70 (d, 5.2)	103.0
5	2.98 (d, 8.0)	39.7		72.7	2.66, overlap	39.2	2.91 (d, 8.0)	39.7
6	4.97 (d, 4.4)	79.4	4.34 (d, 4.0)	79.2	4.21 (d, 4.8)	79.3	4.71, overlap	79.4
7	2.35 (brd, 15.2)	46.0	1.96 (m)	44.3	2.07 (d, 15.6)	46.2	2.12 (d, 15.6)	46.1
	2.19 (brd, 15.2)		1.67 (m)		1.80 (dd, 4.8, 15.6)		2.0 (m)	
8		89.9		88.2		89.6		89.8
9	2.81 (d, 8.4)	50.1	2.79 (s)	55.8	2.66, overlap	49.9	3.35, overlap	49.8
10	1.58 (s)	22.5	1.38 (s)	22.1	1.45 (s)	22.5	1.49 (s)	22.4
COOCH_3_		172.9		172.9		172.9		173.0
COOCH_3_	1.99 (s)	22.2	1.96 (s)	22.2	1.93 (s)	22.3	1.94 (s)	22.2
1′	5.47 (d, 4.4)	96.3	5.53 (d, 2.4)	95.3	5.52 (brd, 1.8)	95.5	5.48 (d, 2.8)	96.4
3′	7.42 (s)	153.0	7.48 (s)	152.4	7.30 (s)	152.0	7.32 (s)	152.4
4′		113.6		114.7		114.7		114.5
5’	2.91 (brdd, 14.4, 8.0)	34.4	2.84 (m)	33.0	2.81 (td, 8.4, 3.6)	32.6	2.83 (td, 8.4, 4.4)	32.9
6′	1.56, overlap	32.5	1.86 (m)	32.2	1.87 (m)	32.2	1.92 (m)	32.4
	2.07 (m)		1.79 (m)		1.61 (m)		1.60 (m)	
7′	1.38 (m)	33.2	1.23 (m)	34.3	1.21 (m)	34.3	1.26 (m)	34.0
	1.79 (m)		1.72 (m)		1.72 (m)		1.73 (m)	
8′	2.28 (m)	37.6	2.24 (m)	36.4	2.23 (m)	36.2	2.25 (m)	36.7
9′	2.27 (m)	44.4	2.34 (td, 8.8, 2.0)	44.2	2.36 (td, 9.6, 1.8)	44.1	2.34 (td, 8.8, 2.8)	44.3
10′	1.08 (d, 6.8)	16.6	1.00 (d, 7.2)	16.5	0.98 (d, 7.2)	16.5	1.01 (d, 6.8)	16.4
11′		168.4		168.1		167.9		167.9
1″	4.68 (d, 8.0)	99.8	4.57 (d, 8.0)	99.9	4.63 (d, 7.8)	99.8	4.65 (d, 8.0)	99.9
2″	3.21 (m)	74.7	3.20 (t, 8.4)	74.4	3.20 (t, 8.4)	74.7	3.20 (t, 8.0)	74.7
3″	3.37 (m)	78.0	3.37 (m)	77.6	3.38, overlap	77.9	3.37, overlap	78.0
4″	3.31, overlap	71.6	3.30, overlap	71.5	3.31, overlap	71.6	3.27, overlap	71.6
5″	3.31, overlap	78.2	3.30, overlap	78.1	3.39, overlap	78.1	3.35, overlap	78.2
6″	3.85 (m)	62.9	3.91 (dd, 12.0)	62.7	3.88 (d, 12.0)	62.8	3.89 (dd)	62.9
	3.65 (m)		3.69 (dd, 5.2, 11.6)		3.69 (m)		3.70 (d, 5.2)	
1′′′	4.64 (d, 8.0)	99.8	4.86, overlap	96.7	4.85, overlap	97.0	4.81 (d, 8.0)	97.9
2′′′	3.19 (m)	74.8	4.79 (d, 8.4)	74.6	4.79 (t, 8.4)	74.6	4.77 (t, 8.4)	74.3
3′′′	3.37 (m)	78.0	3.60 (t, 8.8)	75.9	3.62 (t, 9.0)	75.8	3.55 (t, 8.8)	75.9
4′′′	3.26, overlap	71.7	3.37 (m)	71.8	3.38, overlap	71.7	3.36, overlap	71.8
5′′′	3.31, overlap	78.4	3.37 (m)	78.5	3.38, overlap	78.4	3.36, overlap	78.5
6′′′	3.89 (m)	62.9	3.91 (dd, 12.0)	62.8	3.94 (d,10.8)	62.7	3.94 (d)	62.7
	3.69 (m)		3.69 (dd, 5.2, 11.6)		3.69 (m)		3.67 (d, 5.6)	
1′′′′				127.8		127.2		127.5
2′′′′			7.06 (d, 2.0)	115.8	7.47 (d, 8.4)	131.4	7.70 (d, 8.4)	134.2
3′′′′				146.6	6.83 (d, 8.4)	116.9	6.74 (d, 8.4)	115.7
4′′′′				149.5		161.2		160.1
5′′′′			6.79 (d, 8.0)	116.4	6.83 (d, 8.4)	116.9	6.74 (d, 8.4)	115.7
6′′′′			6.97 (dd, 1.6, 8.0)	123.3	7.47 (d, 8.4)	131.4	7.70 (d, 8.4)	134.2
7′′′′			7.49 (d, 16.0)	147.1	7.56 (d, 15.6)	146.5	6.87 (d, 12.8)	145.9
8′′′′			6.20 (d, 16.0)	114.9	6.26 (d, 15.6)	115.0	5.70 (d, 12.8)	116.2
9′′′′				168.3		168.1		166.8

^a^ Compound **1**, **2**, **4**: Measured at ^1^H (400 MHz) and ^13^C (100 MHz), CD_3_OD; ^b^ Compound **3**: Measured at ^1^H (600 MHz), and ^13^C (150 MHz), CD_3_OD. brd: broad doublet; brdd: broad doublet of doublets; d: doublet; dd: doublet of doublets; m: multiplet; s: singlet; t: triplet; td: triplet of doublets.

**Table 2:** ^1^H- and ^13^C-NMR data for **5**, measured at ^1^H (600 MHz), and ^13^C (150 MHz), CD_3_OD.

Position	Compound 5	
	δ_H_ (*J*)	δ_C_
1	5.50 (d, 3.0)	95.6
3	7.53 (s)	153.7
4		113.3
5	2.82 (m)	33.7
6	1.92 (m)	31.7
	1.60 (m)	
7	1.24 (m)	33.7
	1.74 (m)	
8	2.26 (m)	36.5
9	2.27 (m)	44.2
10	1.03 (d, 6.6)	16.6
11		167.0
1′	4.83, overlap	97.2
2′	4.81, overlap	74.7
3′	3.59 (t, 9.0)	75.9
4′	3.37 (m)	71.8
5′	3.37 (m)	78.6
6′	3.76 (dd,1.8,12.0)	62.2
	3.63 (dd,4.8,12.0)	
1″	5.08 (d, 8.4)	95.5
2″	3.32, overlap	73.9
3″	3.36, overlap	77.9
4′′′	3.34, overlap	70.9
5″	3.15 (m)	78.5
6″	3.93 (d, 11.4)	62.8
	3.69 (d, 12.0)	
1′′′		127.4
2′′′	7.52 (d, 8.4)	131.7
3′′′	6.83 (d, 8.4)	116.7
4′′′		161.1
5′′′	6.83 (d, 8.4)	116.7
6′′′	7.52 (d, 8.4)	131.7
7′′′	7.60 (d, 15.6)	147.2
8′′′	6.29 (d, 15.6)	114.9
9′′′		168.5

**Table 3:** The IC_50_ (mM) values of α-glucosidase inhibitory activity of the isolated compounds, **1**–**11**, and acarbose as the control. Each value is shown as a mean ± standard derivation of three replicates.

Compounds	IC_50_	Compounds	IC_50_
**1**	>5.0	**7**	1.38 ± 0.27
**2**	>5.0	**8**	0.82 ± 0.15
**3**	>5.0	**9**	>5.0
**4**	0.38 ± 0.015	**10**	0.33 ± 0.06
**5**	3.35 ± 0.12	**11**	>5.0
**6**	1.89 ± 0.7	acarbose	3.49 ± 0.15

## Supplementary Materials

Supplementary materials can be accessed at: http://www.mdpi.com/1420-3049/21/12/1749/s1.
