# Supplementary material for: New Iridoid Glucosides from Caryopteris incana (Thunb.) Miq. and Their α-Glucosidase Inhibitory Activities
Source: Molecules. 2016 Dec 21;21(12):1749. doi: 10.3390/molecules21121749 (PMC6273012; doi:10.3390/molecules21121749)
Supplement: Supplementary file 1 [file molecules-21-01749-s001.pdf]

Supplementary Materials: New Iridoid Glucosides from *Caryopteris incana* (Thunb.) Miq. and Their α-Glucosidase Inhibitory Activities

Xu-Dong Mao, Gui-Xin Chou, Sen-Miao Zhao and Cheng-Gang Zhang

Contents of Supplementary Information.

No.	Contents	Pages
1.	**Figure S1.** ^{13}C NMR spectrum of caryocanoside B (**1**) in MeOD (100MHz).	2
2.	**Figure S2.** ^{1}H NMR spectrum of caryocanoside B (**1**) in MeOD (400MHz).	3
3.	**Figure S3.** HSQC spectrum of caryocanoside B (**1**) in MeOD.	4
4.	**Figure S4.** HMBC spectrum of caryocanoside B (**1**) in MeOD.	5
5.	**Figure S5.** NOESY spectrum of caryocanoside B (**1**) in MeOD.	6
6.	**Figure S6.** ^{1}H-^{1}H COSY spectrum of caryocanoside B (**1**) in MeOD.	7
7.	**Figure S7.** HRESIMS spectrum of caryocanoside B (**1**).	8
8.	**Figure S8.** IR spectrum of caryocanoside B (**1**).	9
9.	**Figure S9.** ^{13}C NMR spectrum of 5-hydroxy-2‴-*O*-caffeoylcaryocanoside B (**2**) in MeOD (100MHz).	10
10.	**Figure S10.** ^{1}H NMR spectrum of 5-hydroxy-2‴-*O*-caffeoylcaryocanoside B (**2**) in MeOD (400MHz).	11
11.	**Figure S11.** HSQC spectrum of 5-hydroxy-2‴-*O*-caffeoylcaryocanoside B (**2**) in MeOD.	12
12.	**Figure S12.** HMBC spectrum of 5-hydroxy-2‴-*O*-caffeoylcaryocanoside B (**2**) in MeOD.	13
13.	**Figure S13.** NOESY spectrum of 5-hydroxy-2‴-*O*-caffeoylcaryocanoside B (**2**) in MeOD.	14
14.	**Figure S14.** ^{1}H-^{1}H COSY spectrum of 5-hydroxy-2‴-*O*-caffeoylcaryocanoside B (**2**) in MeOD.	15
15.	**Figure S15.** HRESIMS spectrum of 5-hydroxy-2‴-*O*-caffeoylcaryocanoside B (**2**).	16
16.	**Figure S16.** IR spectrum of5-hydroxy-2‴-*O*-caffeoylcaryocanoside B (**2**).	17
17.	**Figure S17.** ^{13}C NMR spectrum of 2‴-*O*-(*E*)-*p*-coumaroyl caryocanoside B (**3**) in MeOD (150MHz).	18
18.	**Figure S18.** ^{1}H NMR spectrum of 2‴-*O*-(*E*)-*p*-coumaroyl caryocanoside B (**3**) in MeOD (600MHz).	19
19.	**Figure S19.** HSQC spectrum of 2‴-*O*-(*E*)-*p*-coumaroyl caryocanoside B (**3**) in MeOD.	20
20.	**Figure S20.** HMBC spectrum of 2‴-*O*-(*E*)-*p*-coumaroyl caryocanoside B (**3**) in MeOD	21
21.	**Figure S21.** NOESY spectrum of 2‴-*O*-(*E*)-*p*-coumaroyl caryocanoside B (**3**) in MeOD.	22
22.	**Figure S22.** ^{1}H-^{1}H COSY spectrum of 2‴-*O*-(*E*)-*p*-coumaroyl caryocanoside B (**3**) in MeOD.	23
23.	**Figure S23.** HRESIMS spectrum of 2‴-*O*-(*E*)-*p*-coumaroyl caryocanoside B (**3**).	24
24.	**Figure S24.** IR spectrum of 2‴-*O*-(*E*)-*p*-coumaroyl caryocanoside B (**3**).	25
25.	**Figure S25.** ^{13}C NMR spectrum of 2‴-*O*-(*Z*)-*p*-coumaroyl caryocanoside B (**4**) in MeOD (100MHz).	25
26.	**Figure S26.** ^{1}H NMR spectrum of 2‴-*O*-(*Z*)-*p*-coumaroyl caryocanoside B (**4**) in MeOD (400MHz).	26
27.	**Figure S27.** HSQC spectrum of 2‴-*O*-(*Z*)-*p*-coumaroyl caryocanoside B (**4**) in MeOD.	27
28.	**Figure S28.** HMBC spectrum of 2‴-*O*-(*Z*)-*p*-coumaroyl caryocanoside B (**4**) in MeOD.	27
29.	**Figure S29.** NOESY spectrum of 2‴-*O*-(*Z*)-*p*-coumaroyl caryocanoside B (**4**) in MeOD.	28
30.	**Figure S30.** ^{1}H-^{1}H COSY spectrum of 2‴-*O*-(*Z*)-*p*-coumaroyl caryocanoside B (**4**) in MeOD.	28
31.	**Figure S31.** HRESIMS spectrum of 2‴-*O*-(*Z*)-*p*-coumaroyl caryocanoside B (**4**).	29
32.	**Figure S32.** IR spectrum of 2‴-*O*-(*Z*)-*p*-coumaroyl caryocanoside B (**4**).	29
33.	**Figure S33.** ^{13}C NMR spectrum of 2′-*O*-(*E*)-*p*-coumaroyl asystasioside A (**5**) in MeOD (150MHz).	30
34.	**Figure S34.** ^{1}H NMR spectrum of 2′-*O*-(*E*)-*p*-coumaroyl asystasioside A (**5**) in MeOD (600MHz).	30
35.	**Figure S35.** HSQC spectrum of 2′-*O*-(*E*)-*p*-coumaroyl asystasioside A (**5**) in MeOD.	31
36.	**Figure S36.** HMBC spectrum of 2′-*O*-(*E*)-*p*-coumaroyl asystasioside A (**5**) in MeOD.	32
37.	**Figure S37.** NOESY spectrum of 2′-*O*-(*E*)-*p*-coumaroyl asystasioside A (**5**) in MeOD.	33
38.	**Figure S38.** ^{1}H-^{1}H COSY spectrum of 2′-*O*-(*E*)-*p*-coumaroyl asystasioside A (**5**) in MeOD.	34
38.	**Figure S39.** HRESIMS spectrum of 2′-*O*-(*E*)-*p*-coumaroyl asystasioside A (**5**).	35
40.	**Figure S40.** IR spectrum of 2′-*O*-(*E*)-*p*-coumaroyl asystasioside A (**5**).	36
41.	**Figure S41.** chemical constitution of compound **1**–**5**.	37
42.	**Figure S42.** chemical constitution of compound **6**–**11**.	38
43.	**Table S1** in vitro α-glucosidase inhibitory activity of compounds **1**–**11**.	38

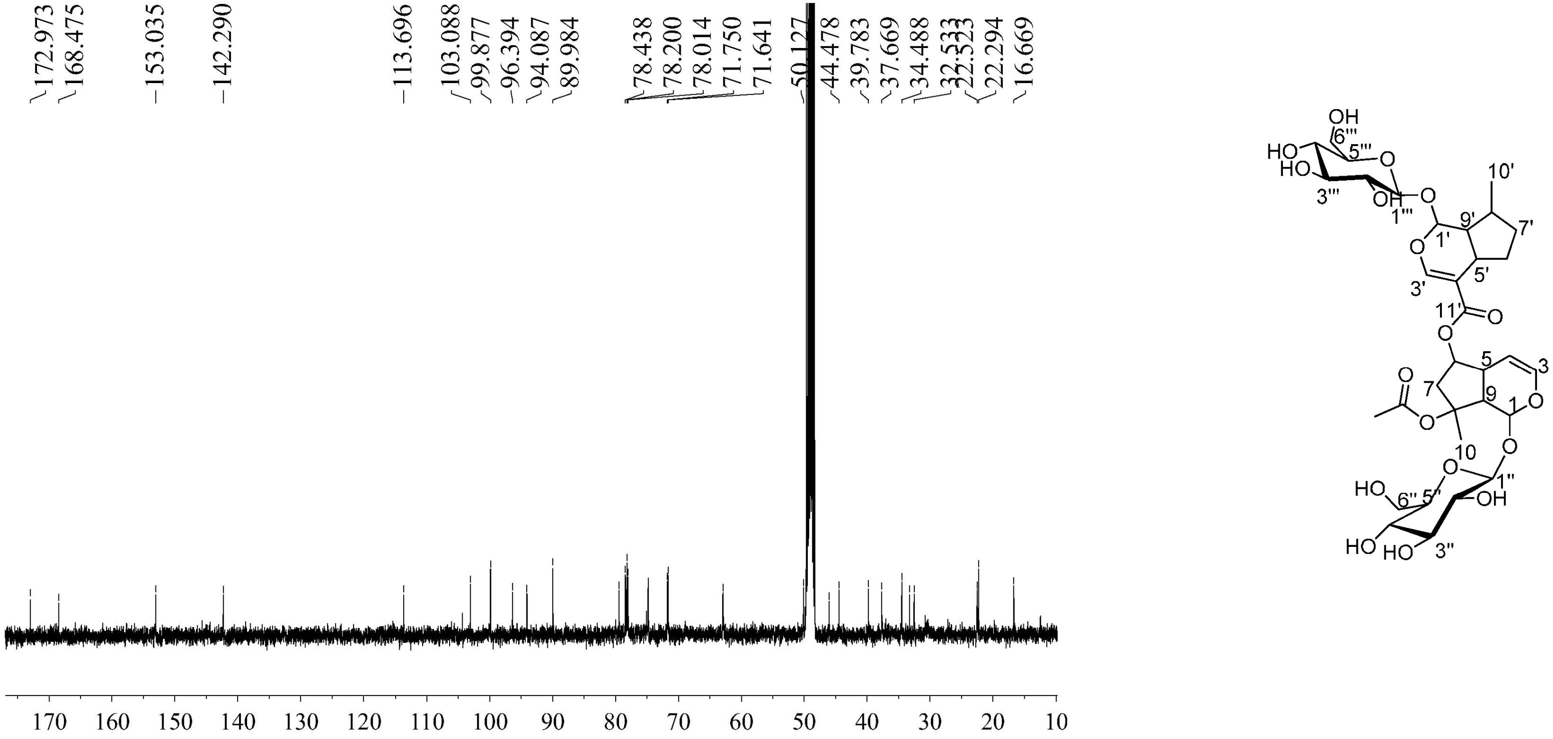

Figure S1. ^{13}C NMR spectrum of caryocanoside B (**1**) in MeOD (100MHz).

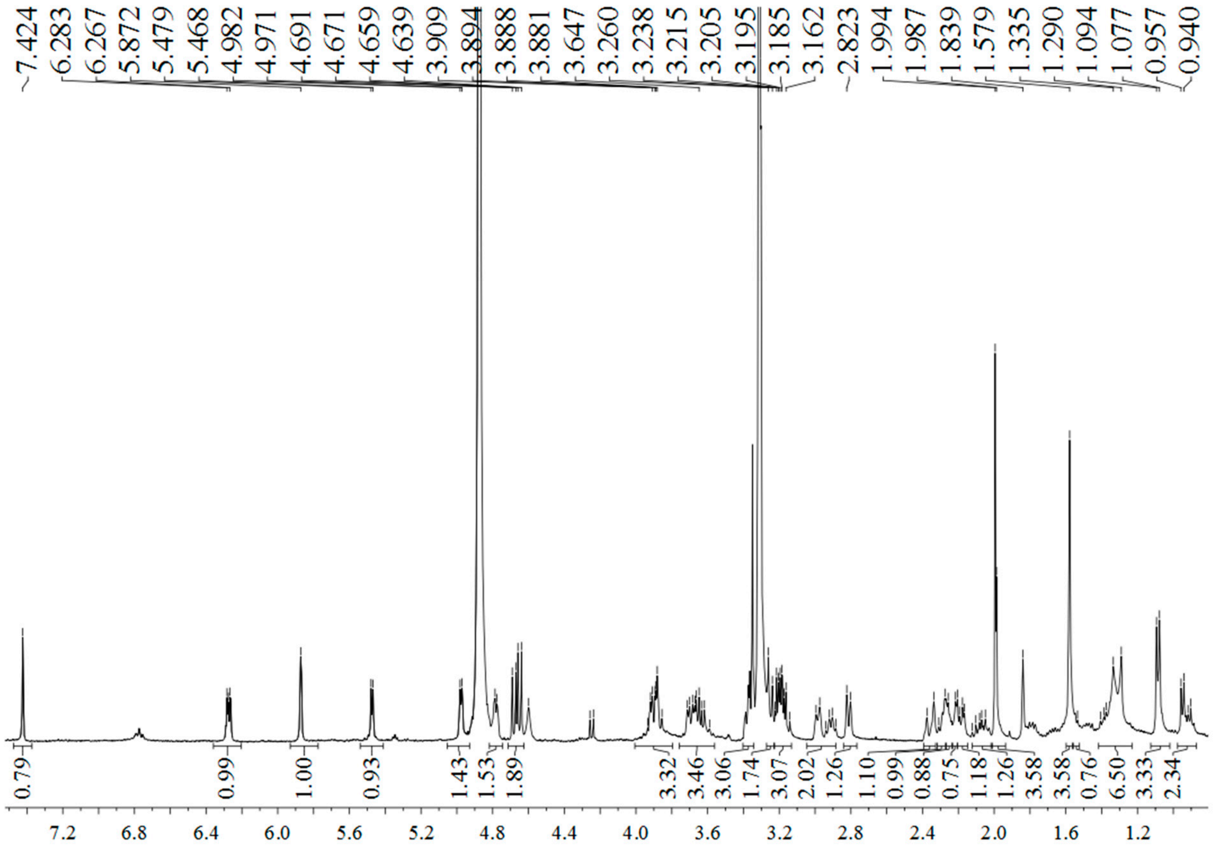

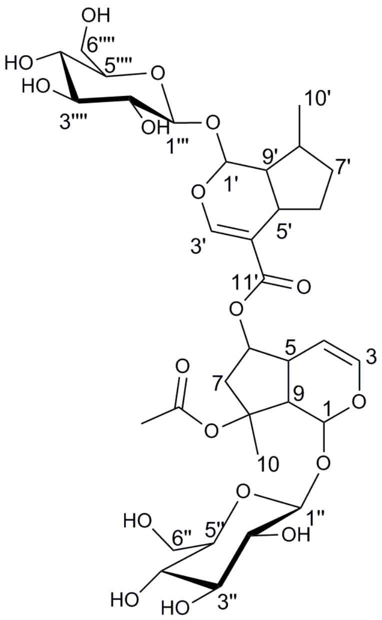

Figure S2. ^{1}H NMR spectrum of caryocanoside B (**1**) in MeOD (400 MHz).

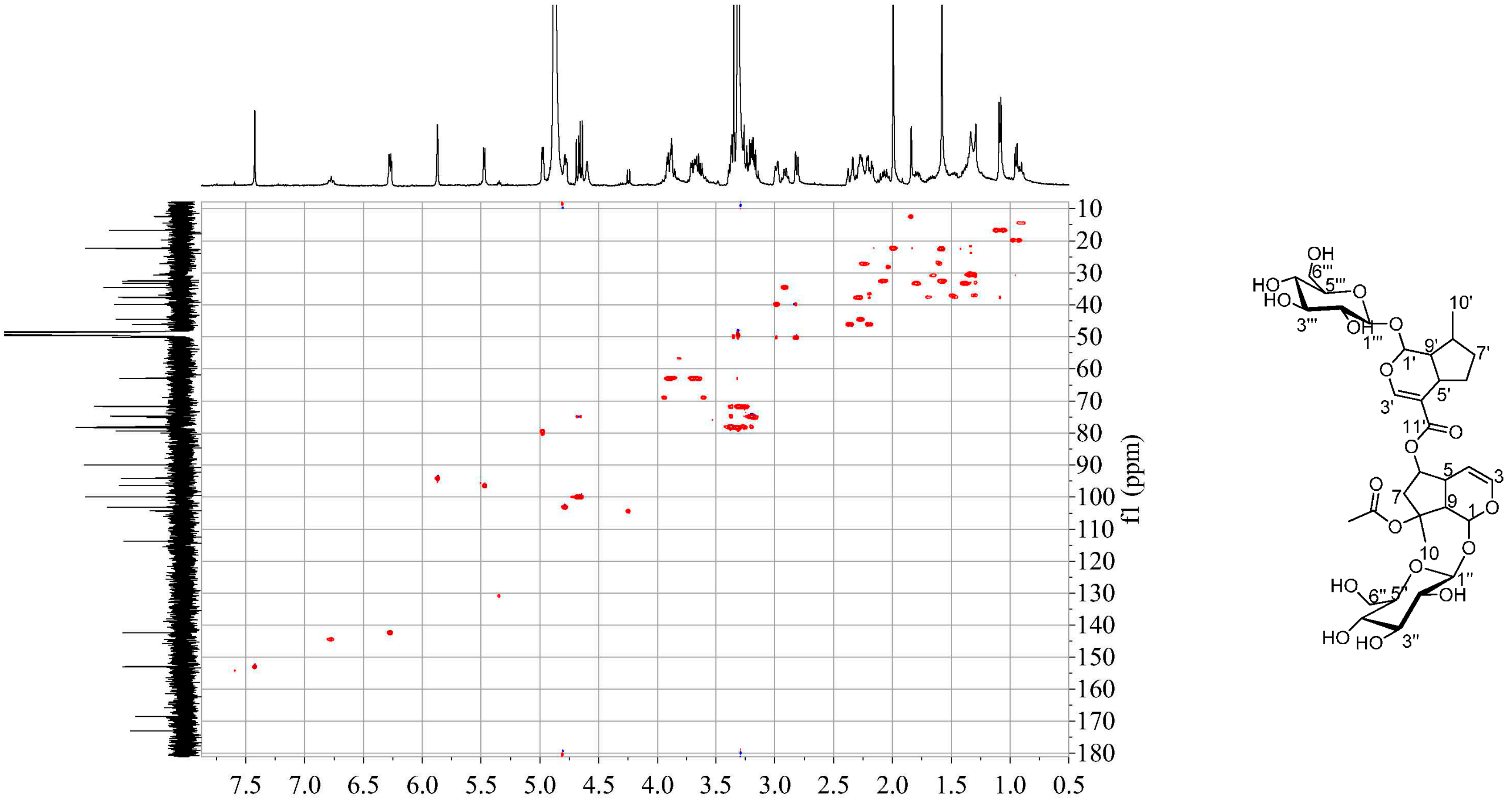

Figure S3. Heteronuclear single quantum correlation (HSQC) spectrum of caryocanoside B (**1**) in MeOD.

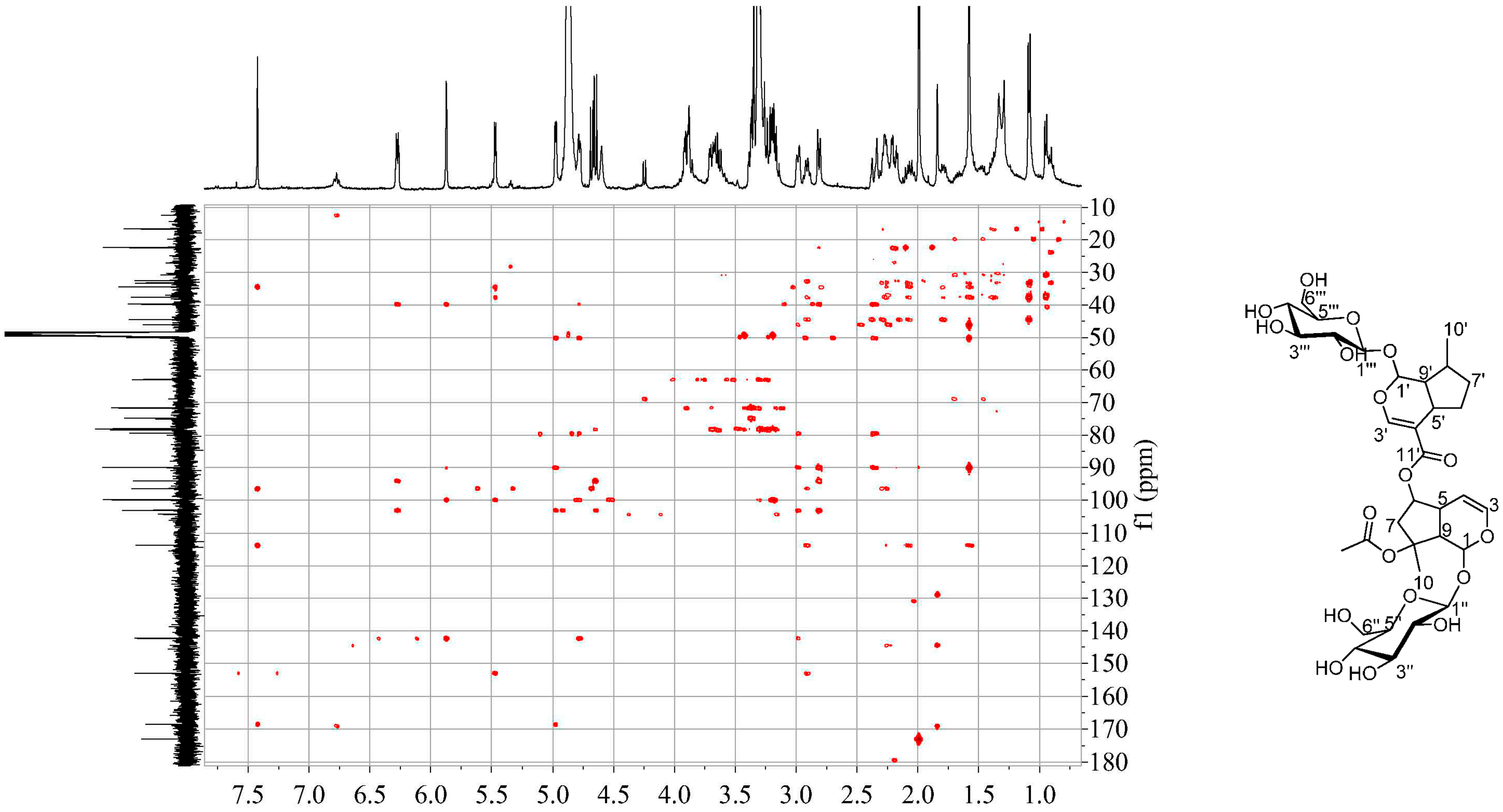

Figure S4. Heteronuclear multiple bond correlation (HMBC) spectrum of caryocanoside B (**1**) in MeOD.

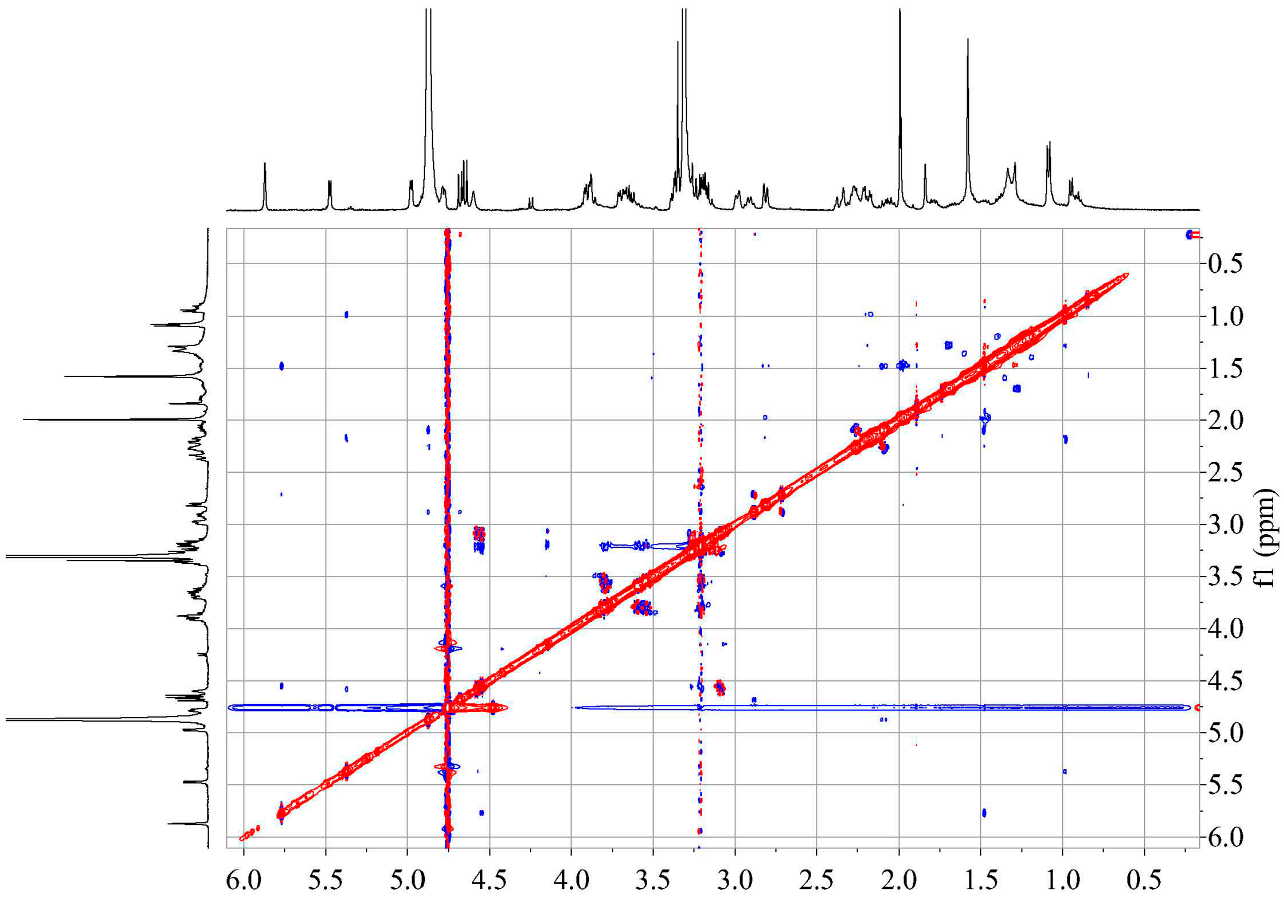

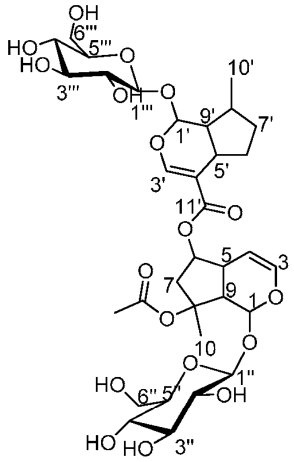

Figure S5. Nuclear Overhauser effect spectroscopy (NOESY) spectrum of caryocanoside B (**1**) in MeOD.

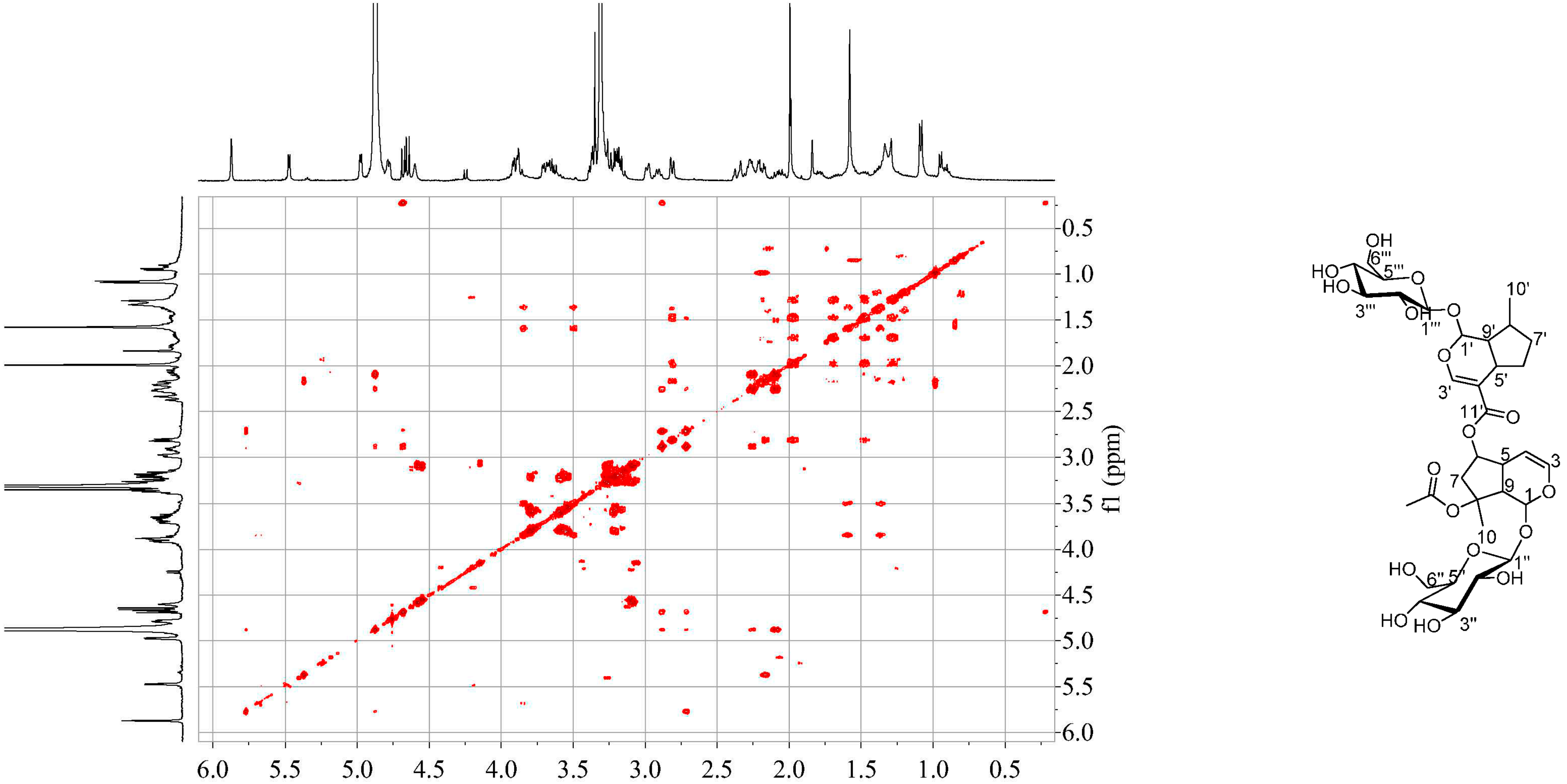

Figure S6. ¹H-¹H correlation spectroscopy (¹H-¹H COSY) spectrum of caryocanoside B (**1**) in MeOD.

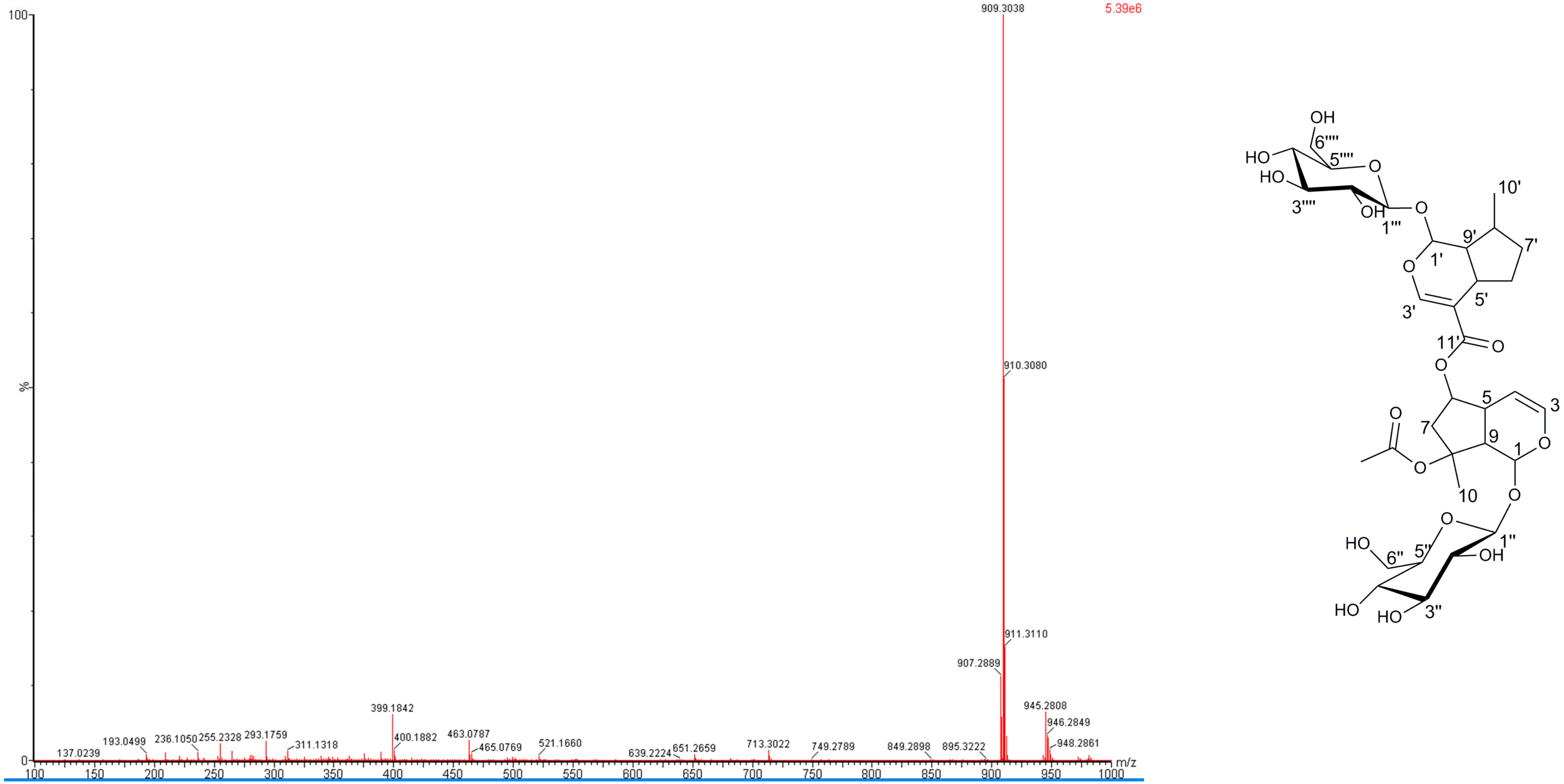

Figure S7. High resolution electrospray ionization mass spectroscopy (HRESIMS) spectrum of caryocanoside B (**1**).

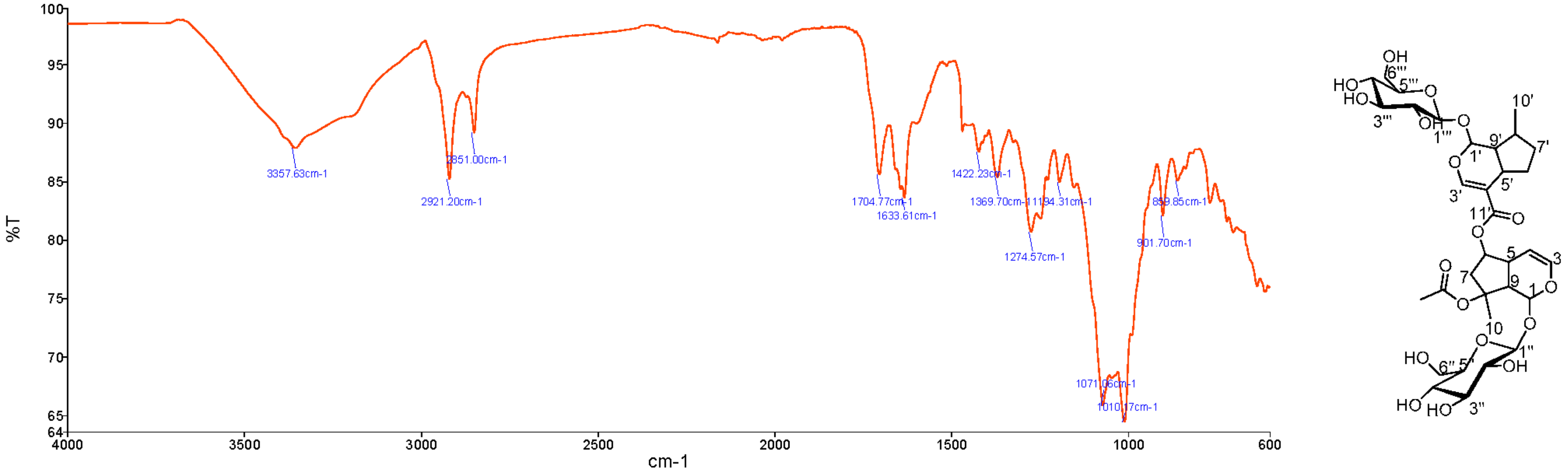

Figure S8. Infrared (IR) spectrum of caryocanoside B (**1**).

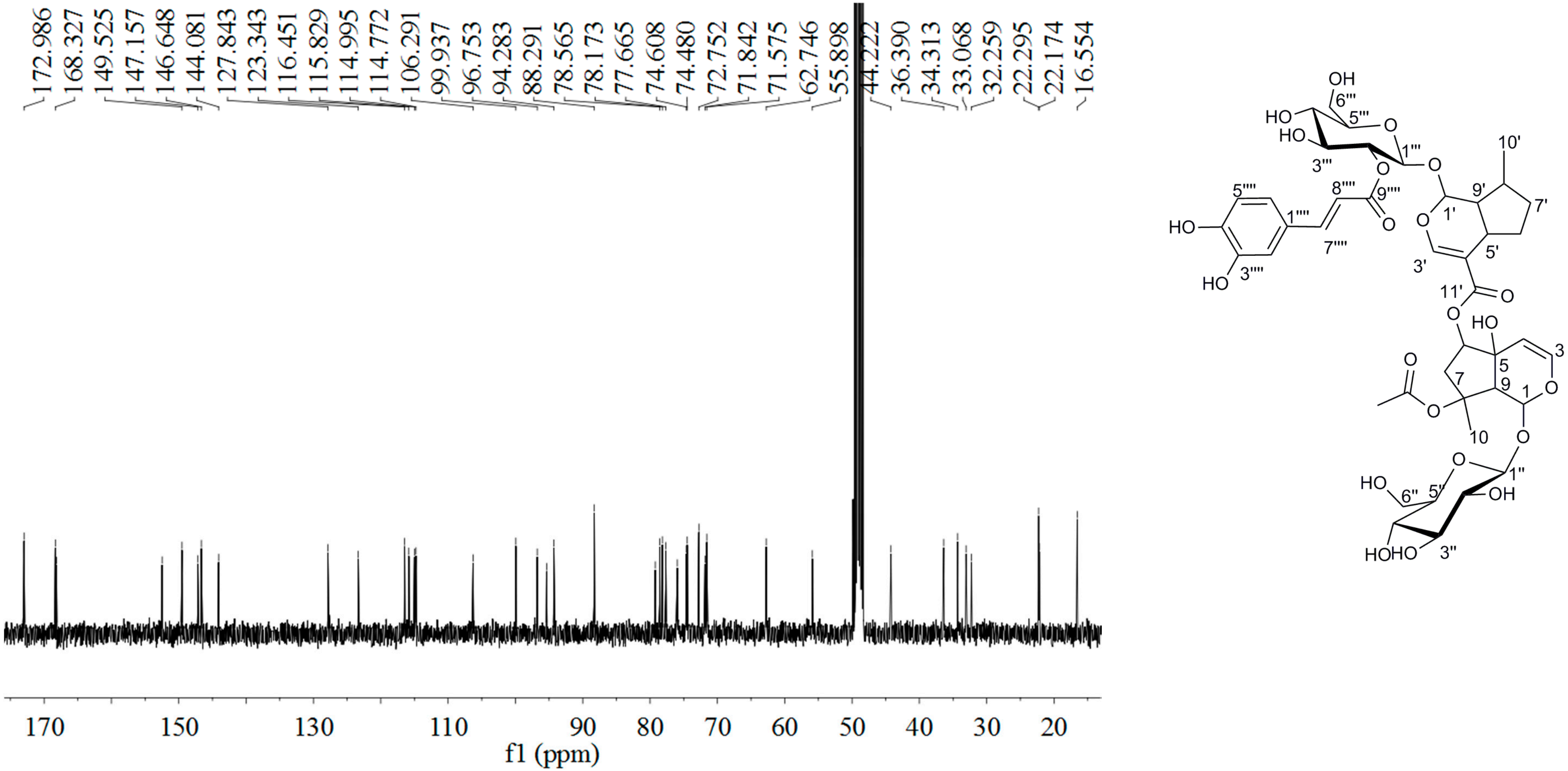

Figure S9. ^{13}C NMR spectrum of 5-hydroxy-2‴-*O*-caffeoylcaryocanoside B (**2**) in MeOD (100 MHz).

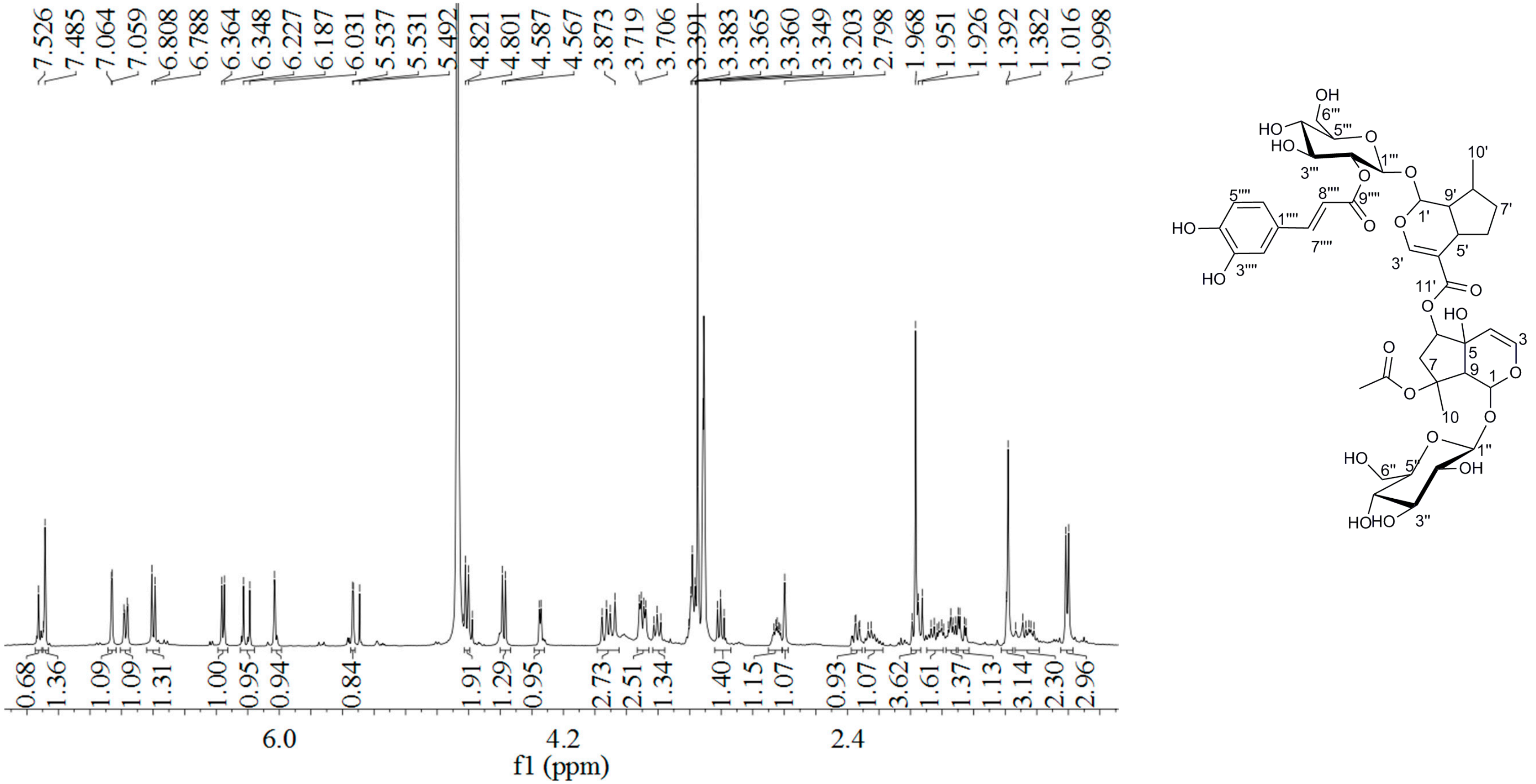

Figure S10. ^{1}H NMR spectrum of 5-hydroxy-2′′′-*O*-caffeoylcaryocanoside B (**2**) in MeOD (400 MHz).

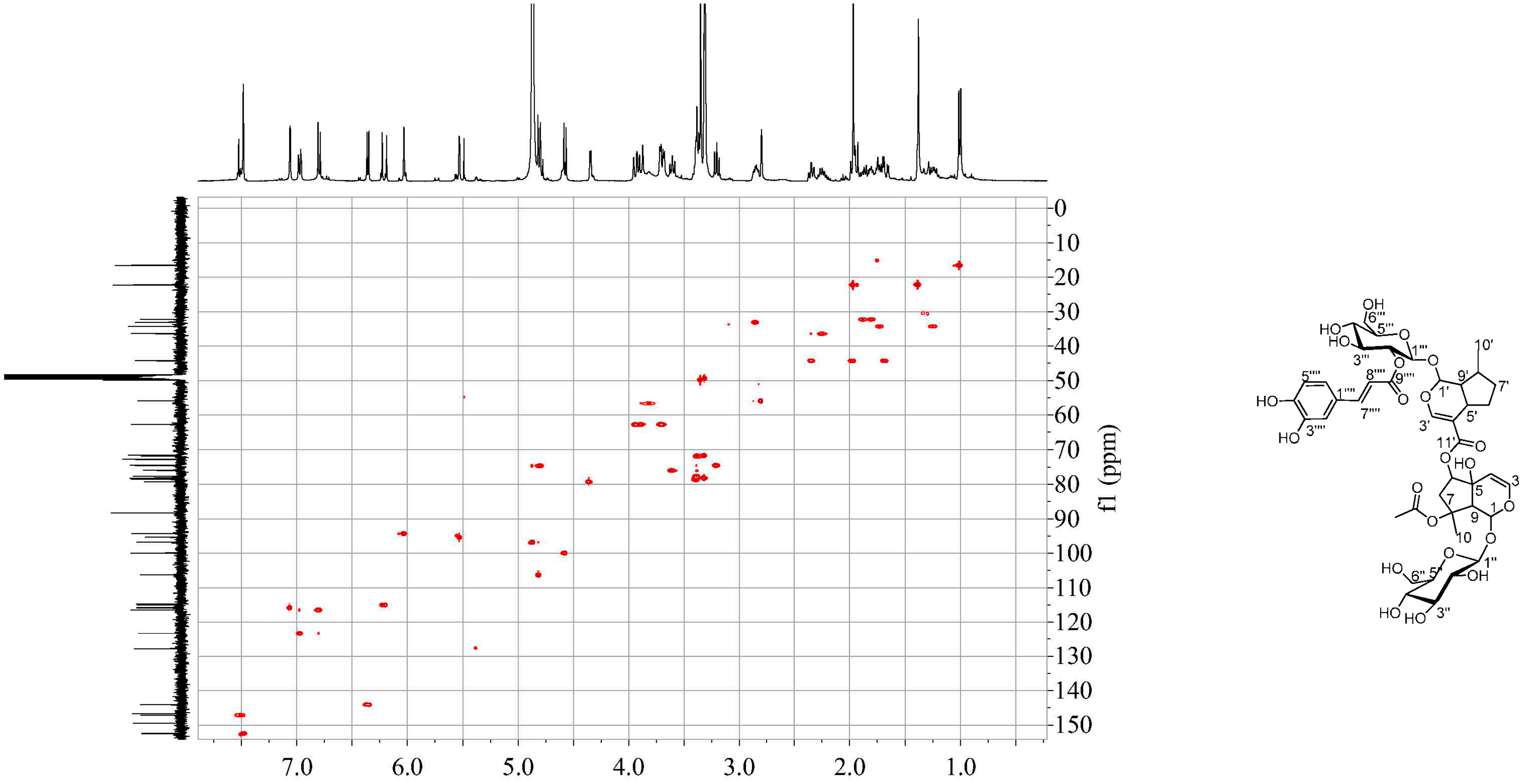

Figure S11. HSQC spectrum of 5-hydroxy-2‴-*O*-caffeoylcaryocanoside B (**2**) in MeOD.

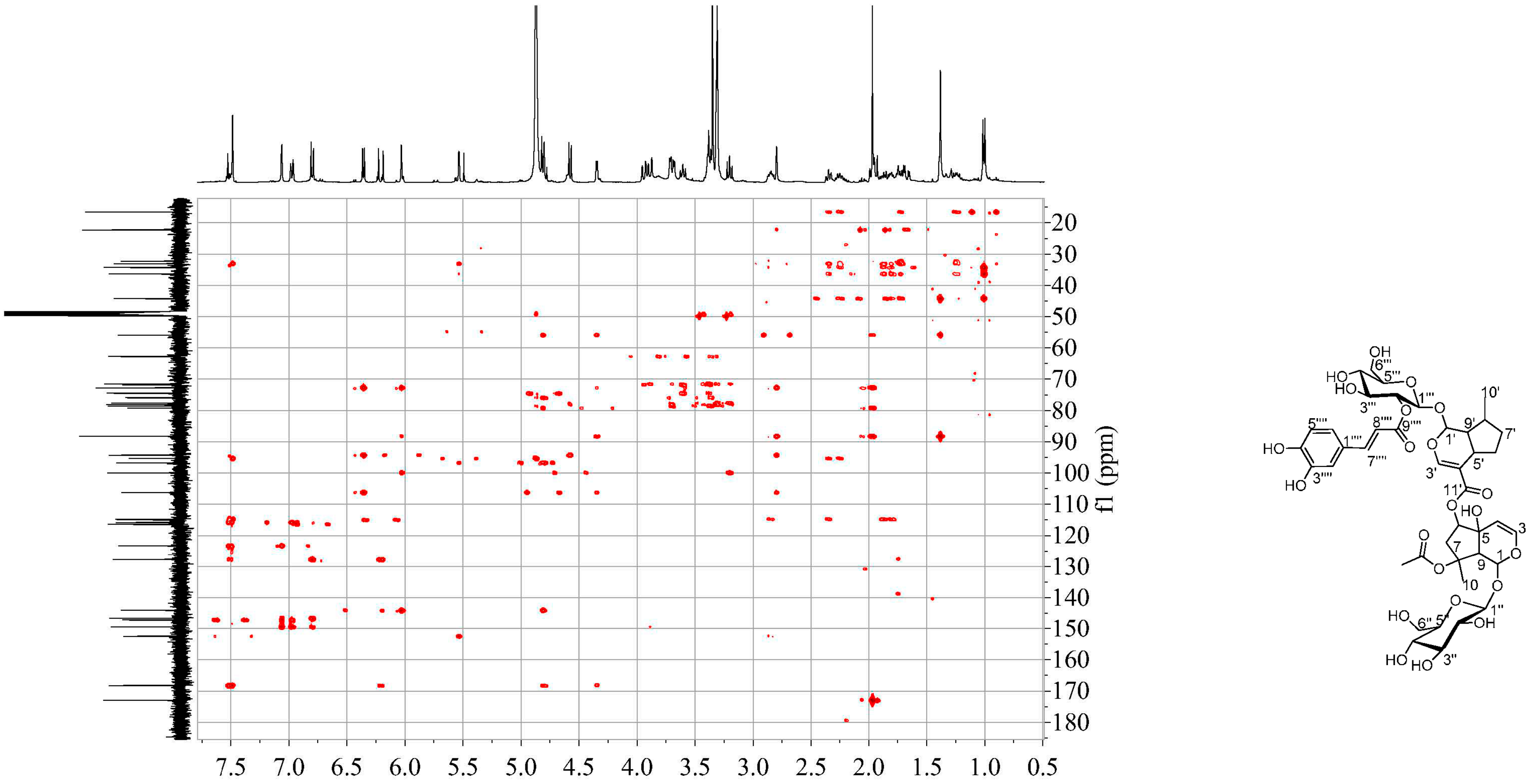

Figure S12. HMBC spectrum of 5-hydroxy-2‴-*O*-caffeoylcaryocanoside B (**2**) in MeOD.

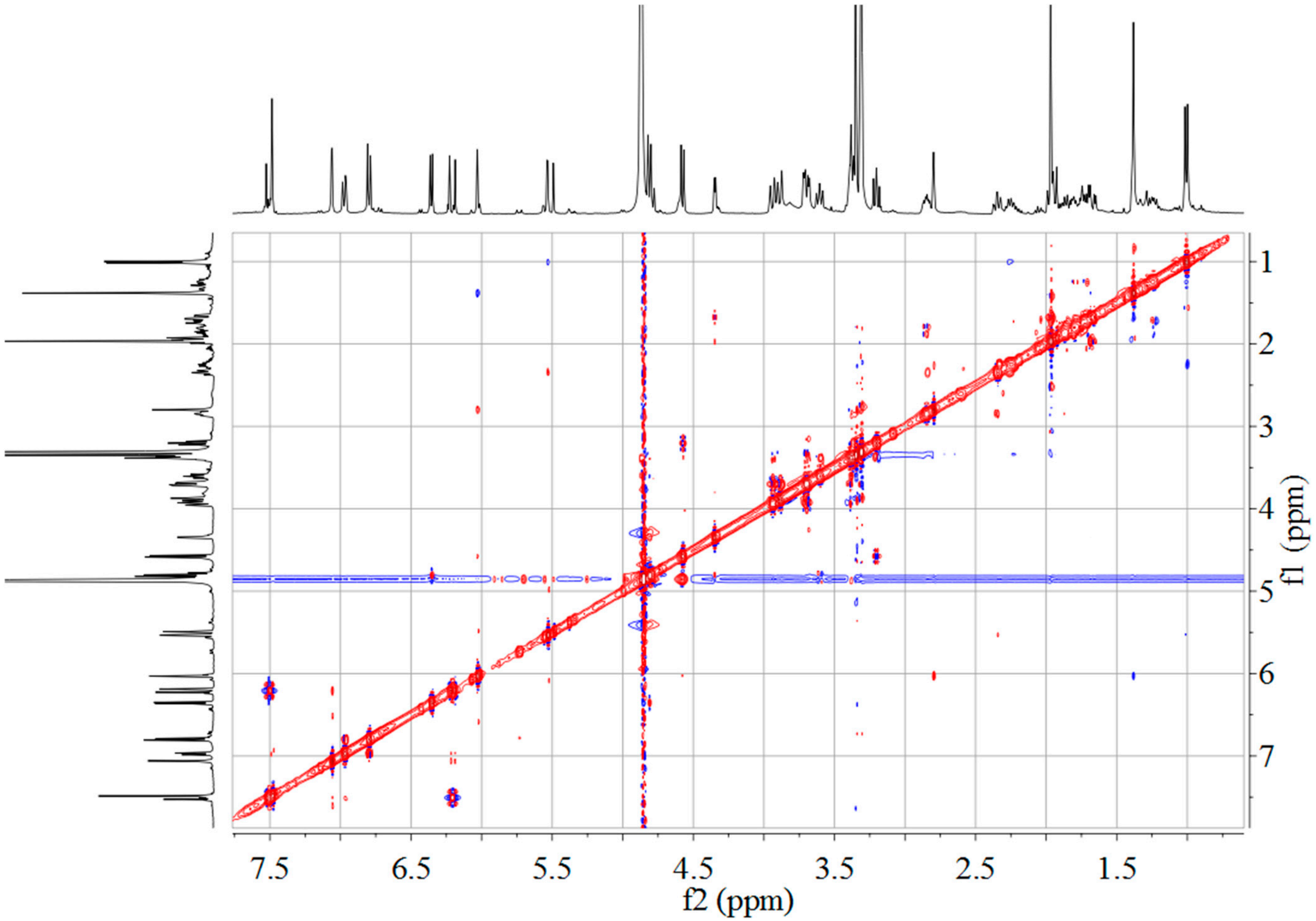

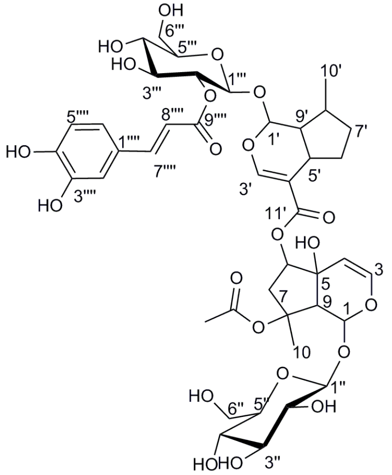

Figure S13. NOESY spectrum of 5-hydroxy-2′′′-*O*-caffeoylcaryocanoside B (**2**) in MeOD.

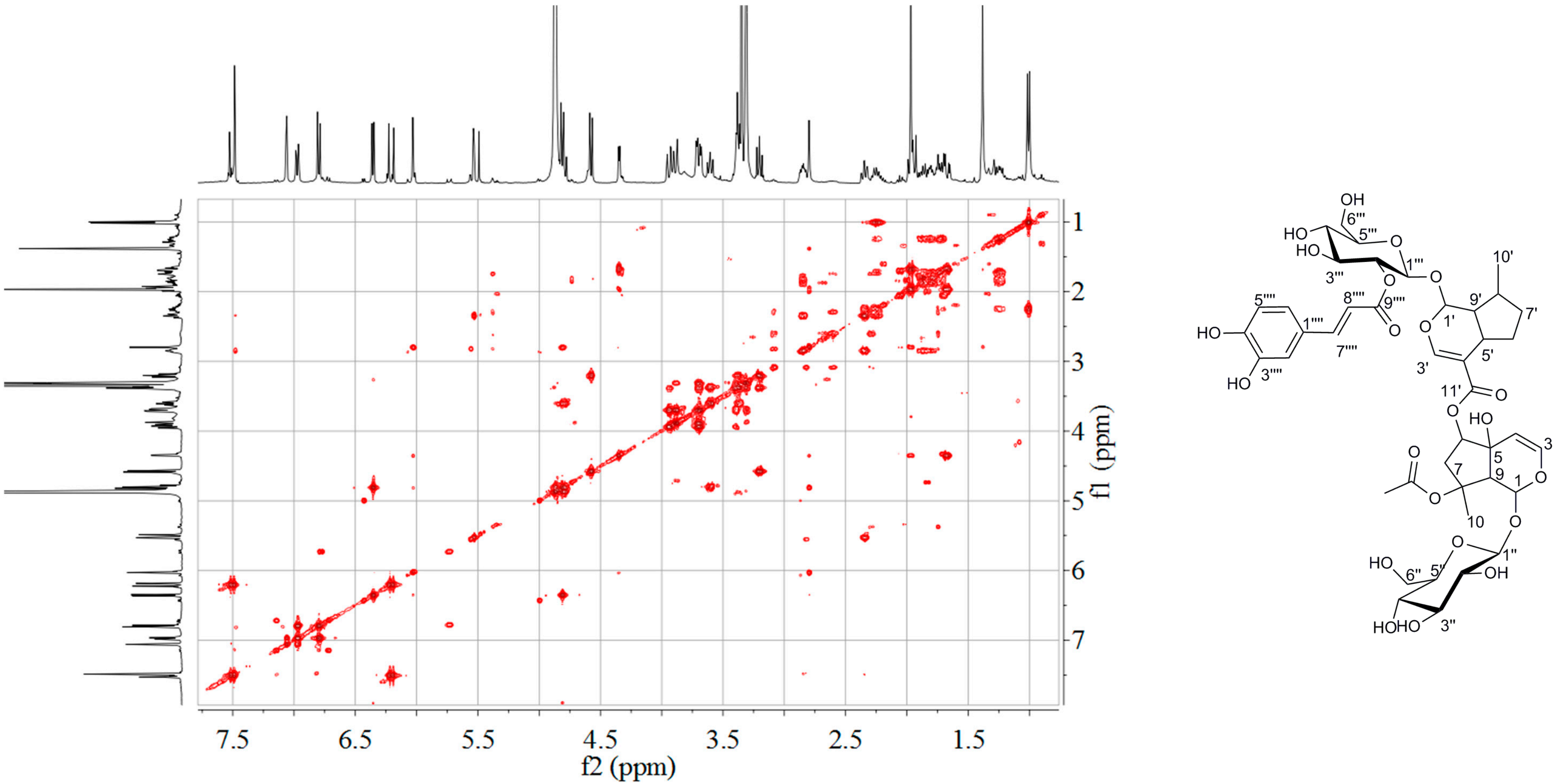

Figure S14. ^{1}H-^{1}H COSY spectrum of 5-hydroxy-2‴-*O*-caffeoylcaryocanoside B (**2**) in MeOD.

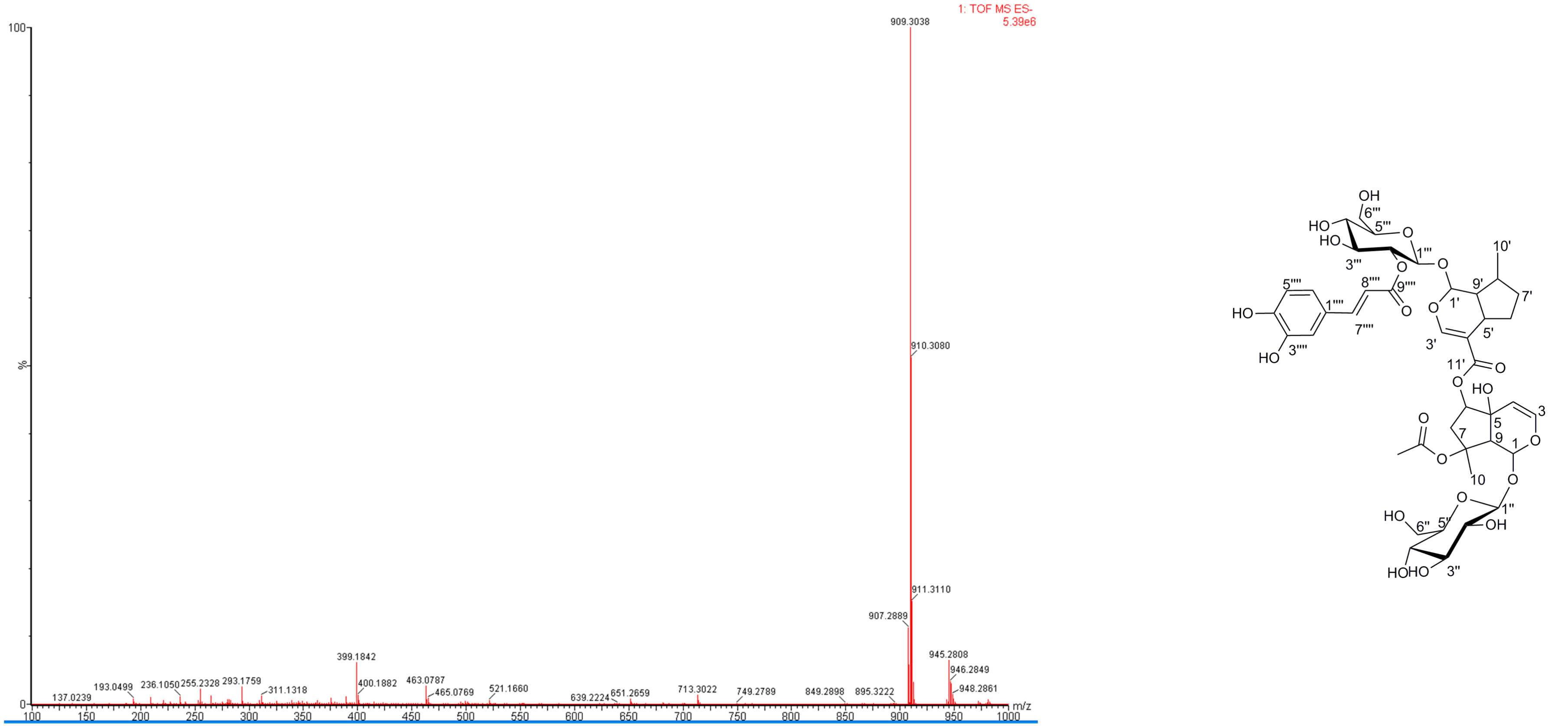

Figure S15. HRESIMS spectrum of 5-hydroxy-2‴-*O*-caffeoylcaryocanoside B (**2**).

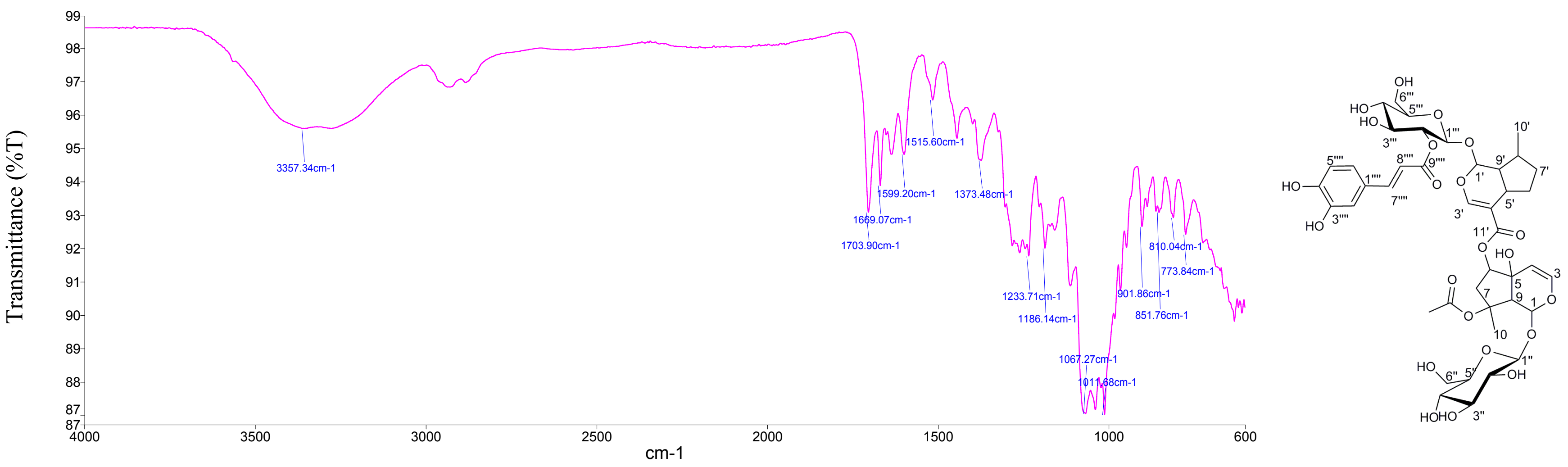

Figure S16. IR spectrum of 5-hydroxy-2‴-*O*-caffeoylcaryocanoside B (**2**).

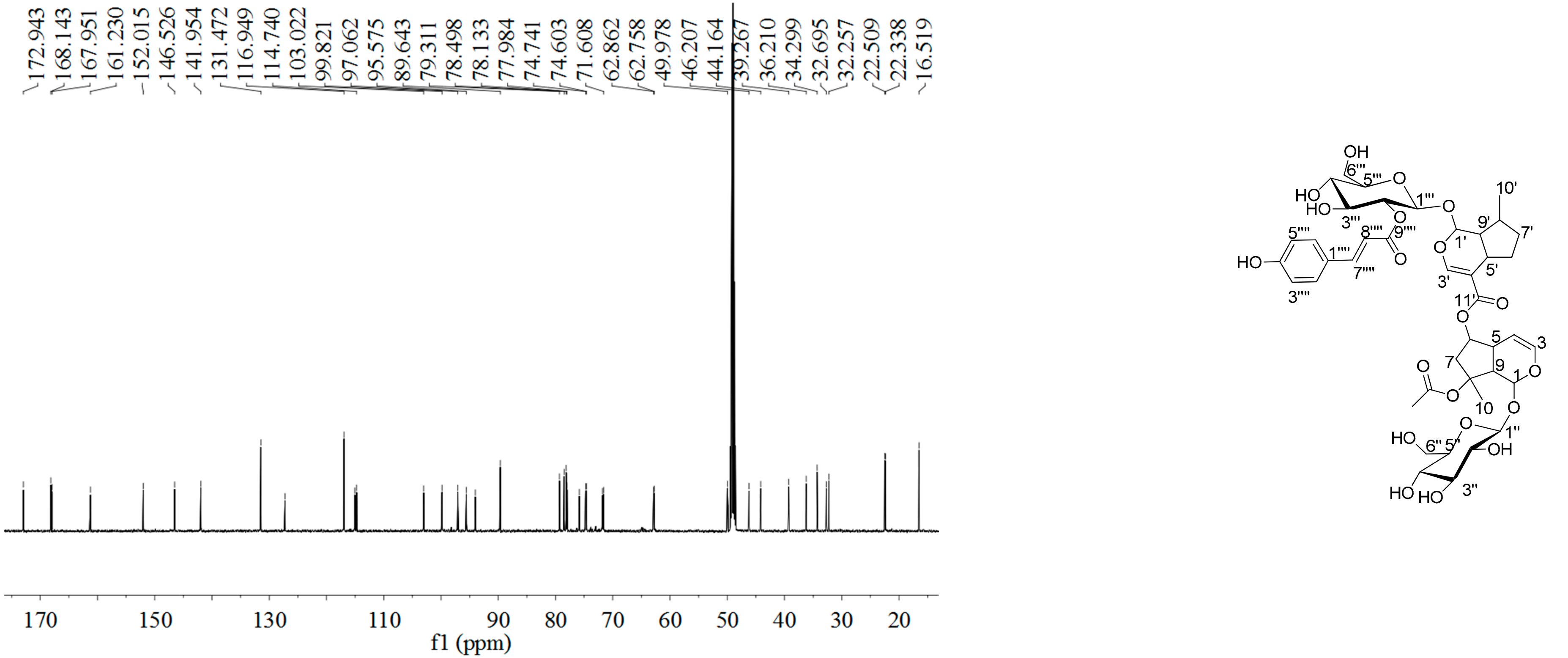

Figure S17. ^{13}C NMR spectrum of 2‴-*O*-(*E*)-*p*-coumaroyl caryocanoside B (**3**) in MeOD (150 MHz).

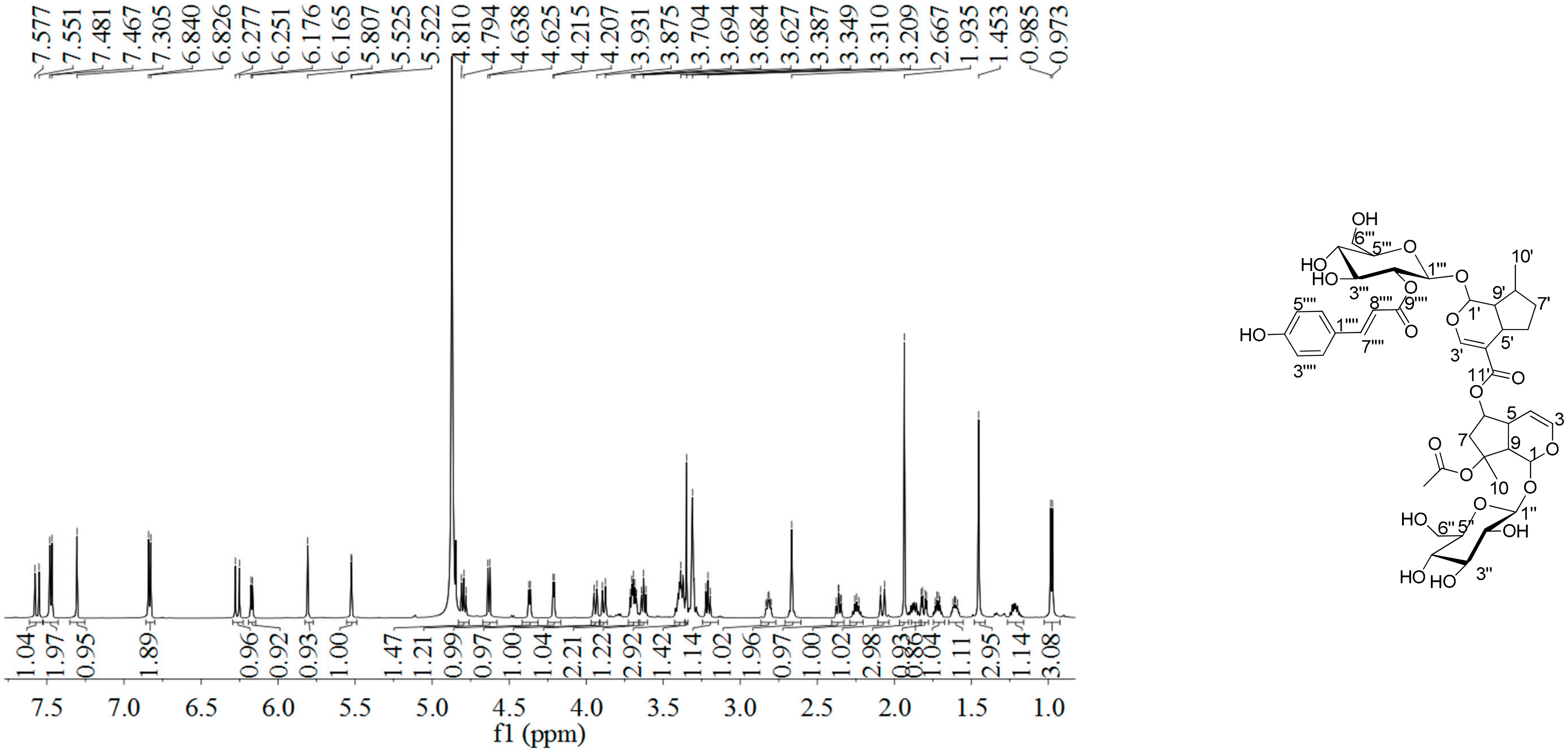

Figure S18. ^{1}H NMR spectrum of 2‴-*O*-(*E*)-*p*-coumaroyl caryocanoside B (**3**) in MeOD (600 MHz).

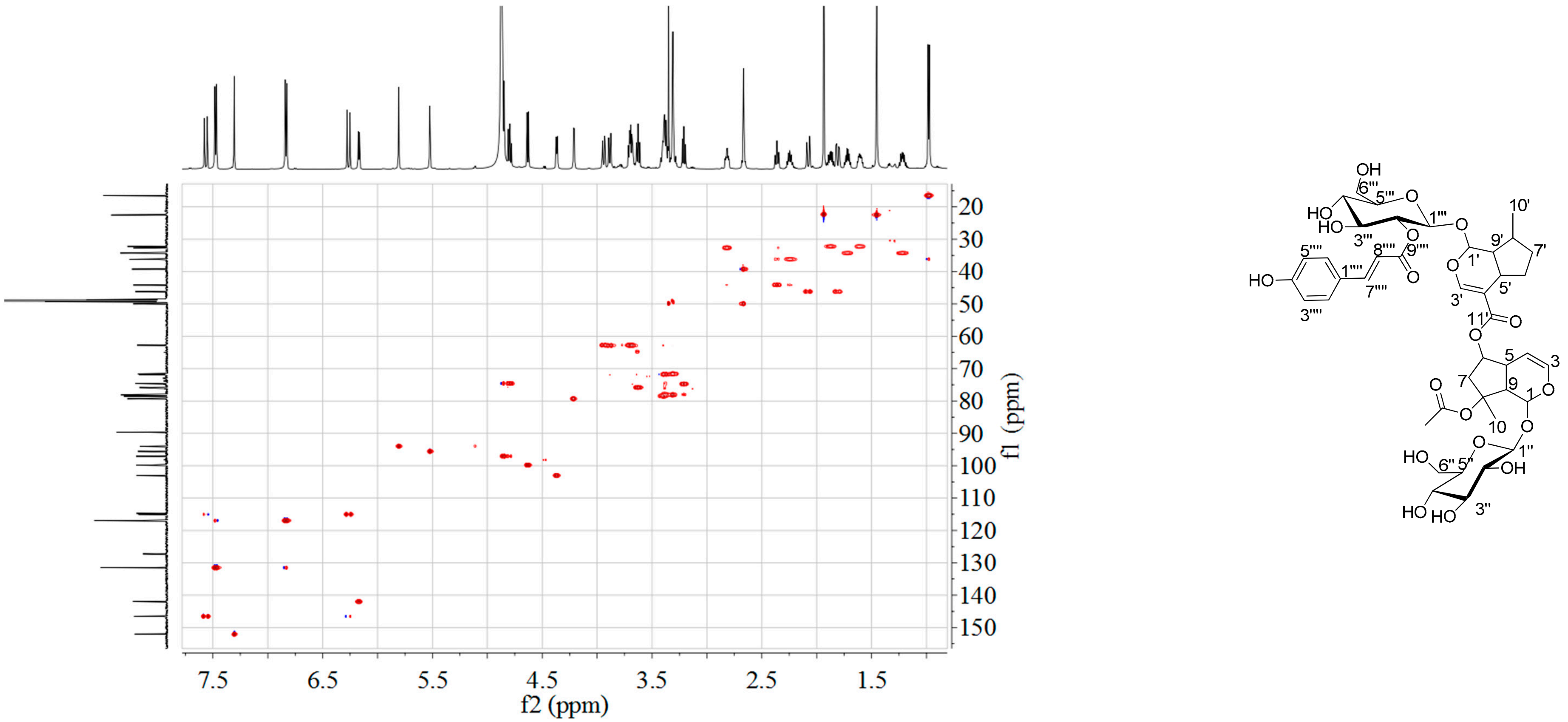

Figure S19. HSQC spectrum of 2′′′-*O*-(*E*)-*p*-coumaroyl caryocanoside B (**3**) in MeOD.

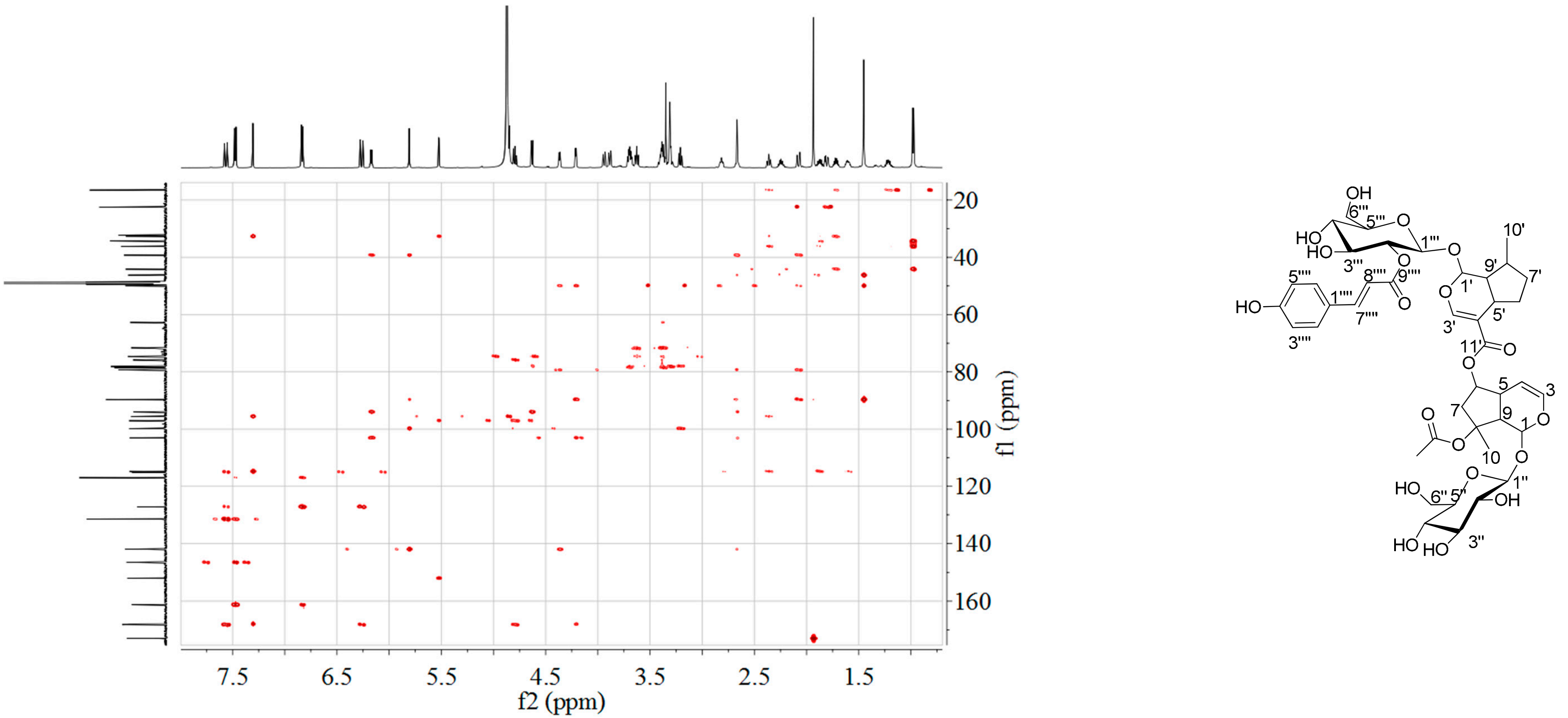

Figure S20. HMBC spectrum of 2'''-*O*-(*E*)-*p*-coumaroyl caryocanoside B (**3**) in MeOD.

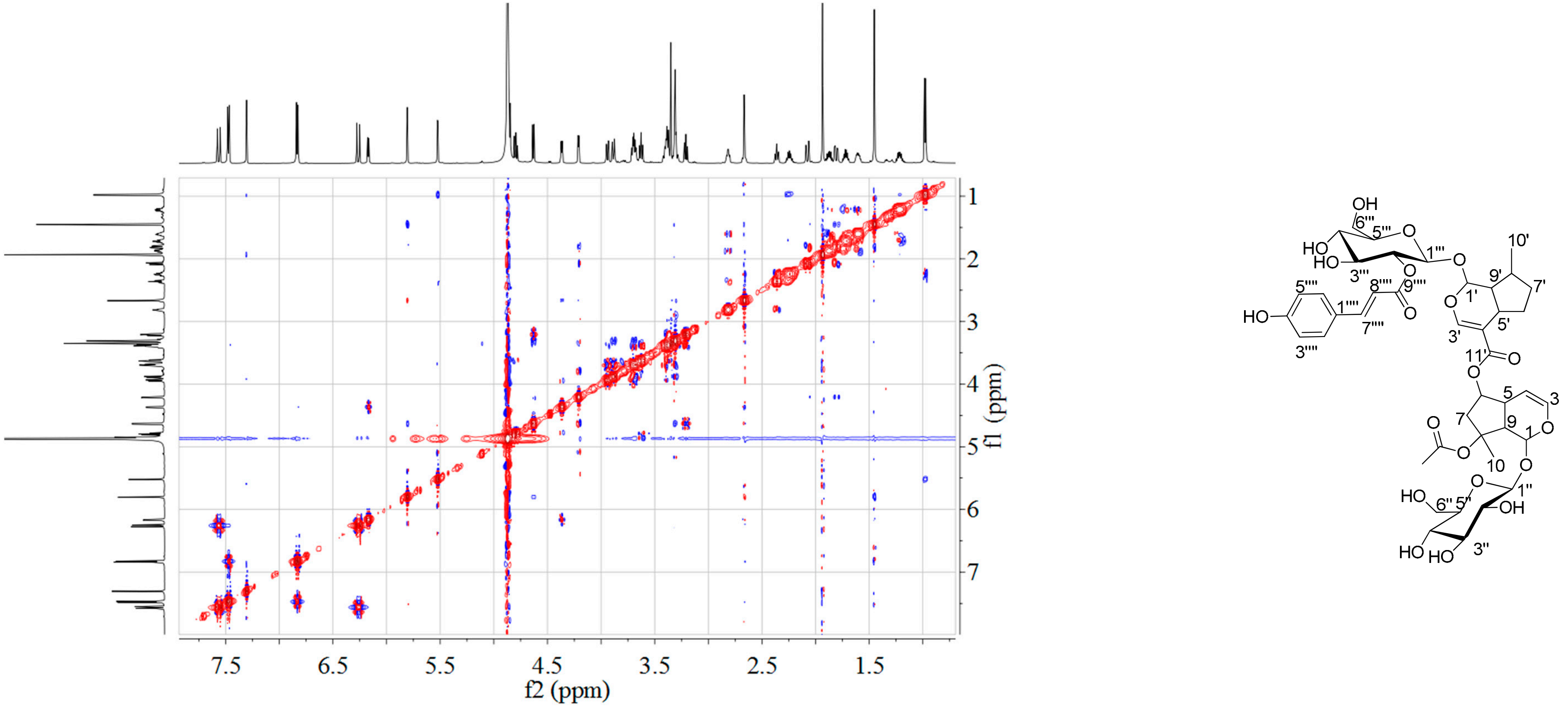

Figure S21. NOESY spectrum of 2‴-*O*-(*E*)-*p*-coumaroyl caryocanoside B (**3**) in MeOD.

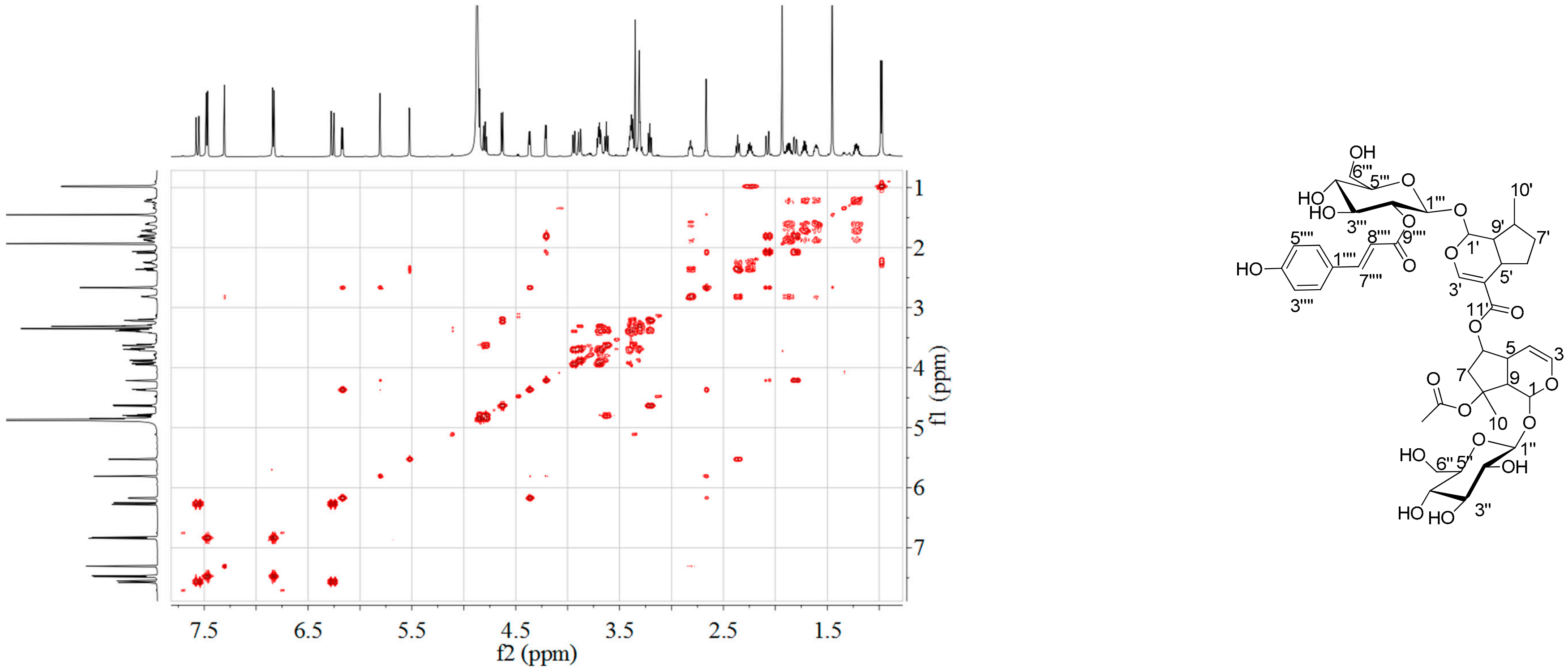

Figure S22. ^{1}H-^{1}H COSY spectrum of 2‴-*O*-(*E*)-*p*-coumaroyl caryocanoside B (**3**) in MeOD.

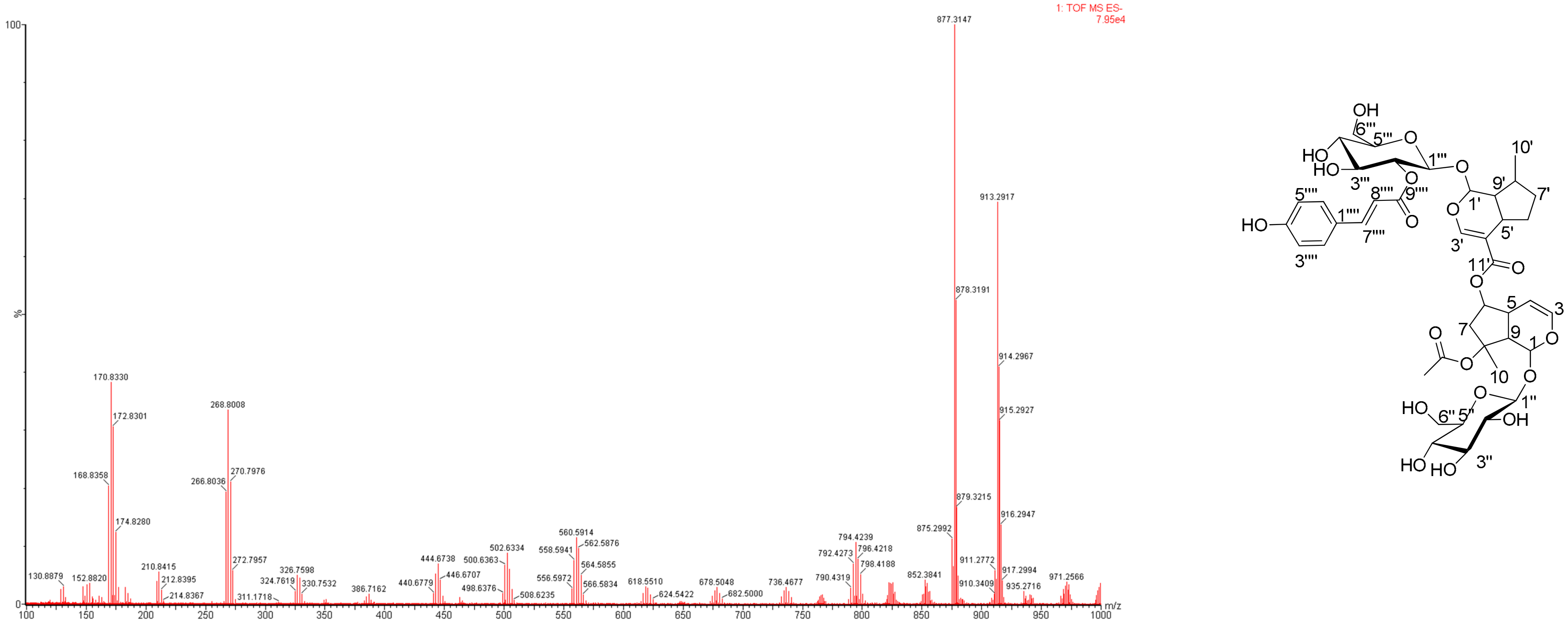

Figure S23. HRESIMS spectrum of 2‴-*O*-(*E*)-*p*-coumaroyl caryocanoside B (**3**).

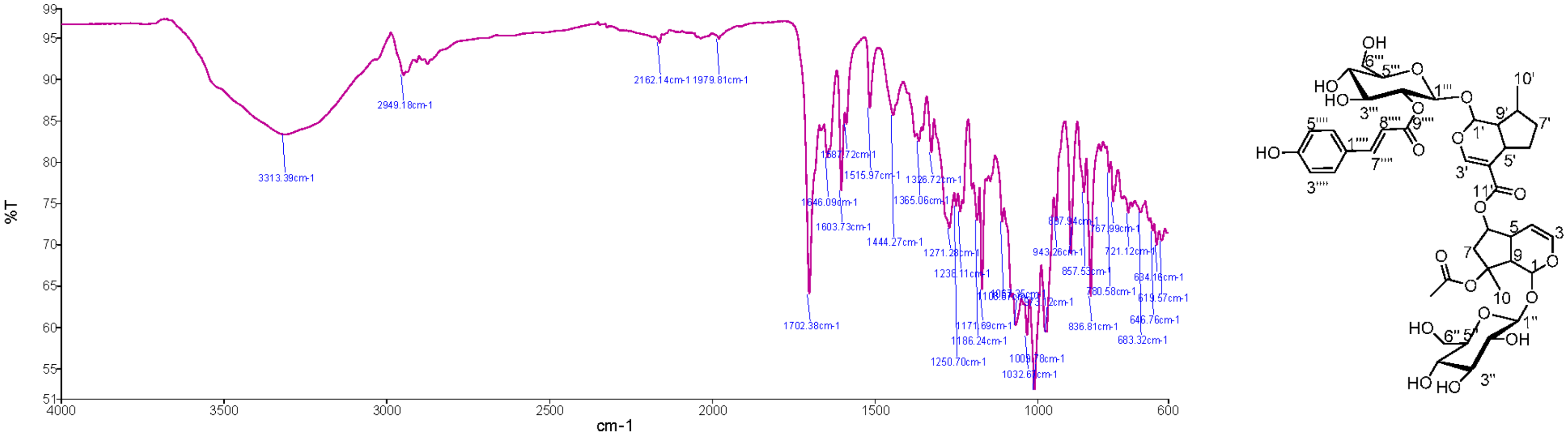

Figure S24. IR spectrum of 2‴-*O*-(*E*)-*p*-coumaroyl caryocanoside B (**3**).

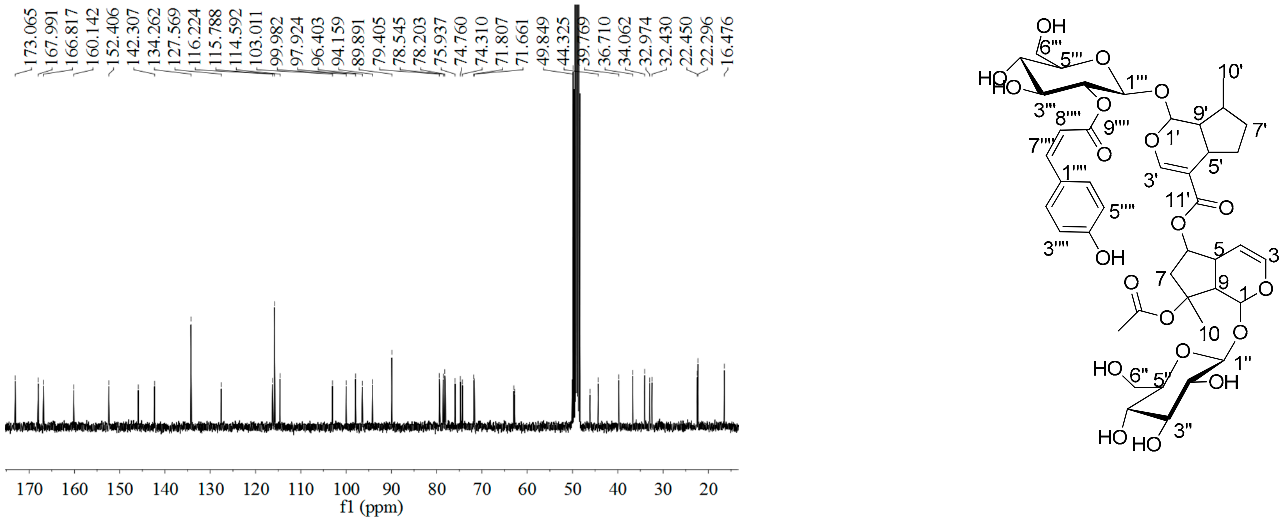

Figure S25. ^{13}C NMR spectrum of 2‴-*O*-(*Z*)-*p*-coumaroyl caryocanoside B (**4**) in MeOD (100 MHz).

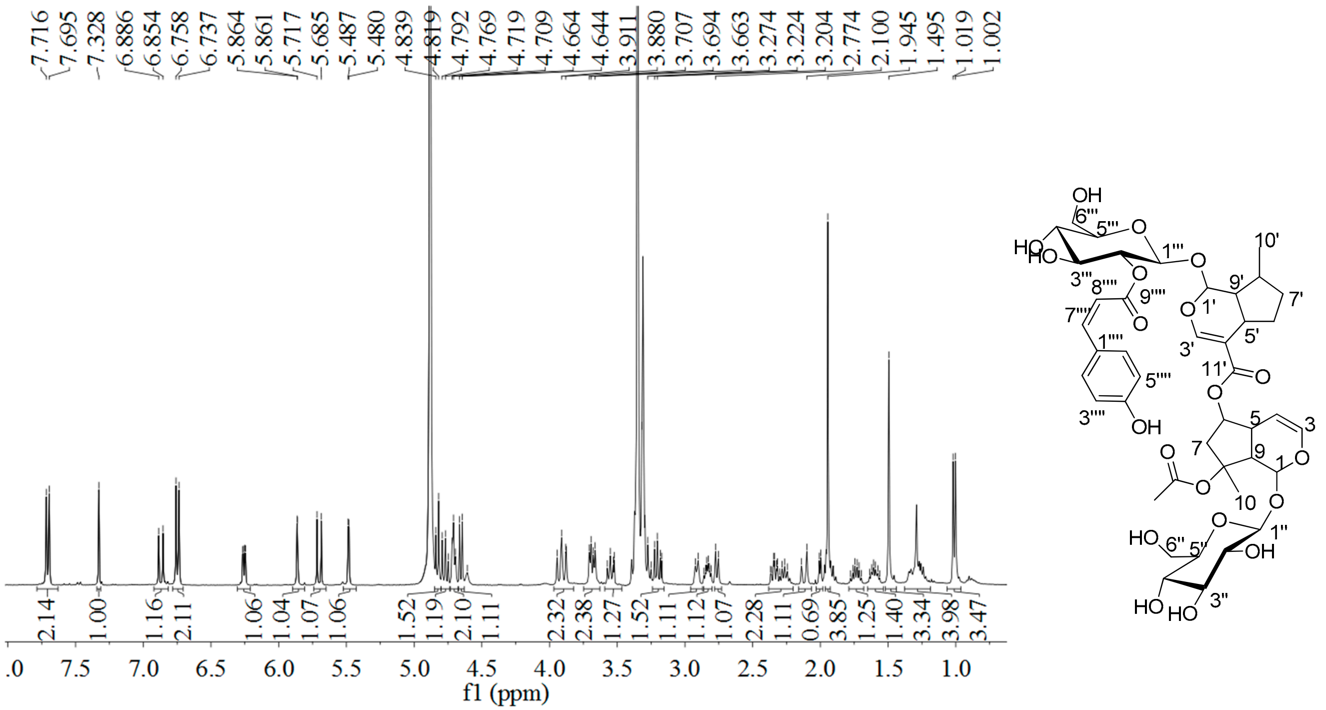

Figure S26. ^{1}H NMR spectrum of 2‴-*O*-(*Z*)-*p*-coumaroyl caryocanoside B (**4**) in MeOD (400 MHz).

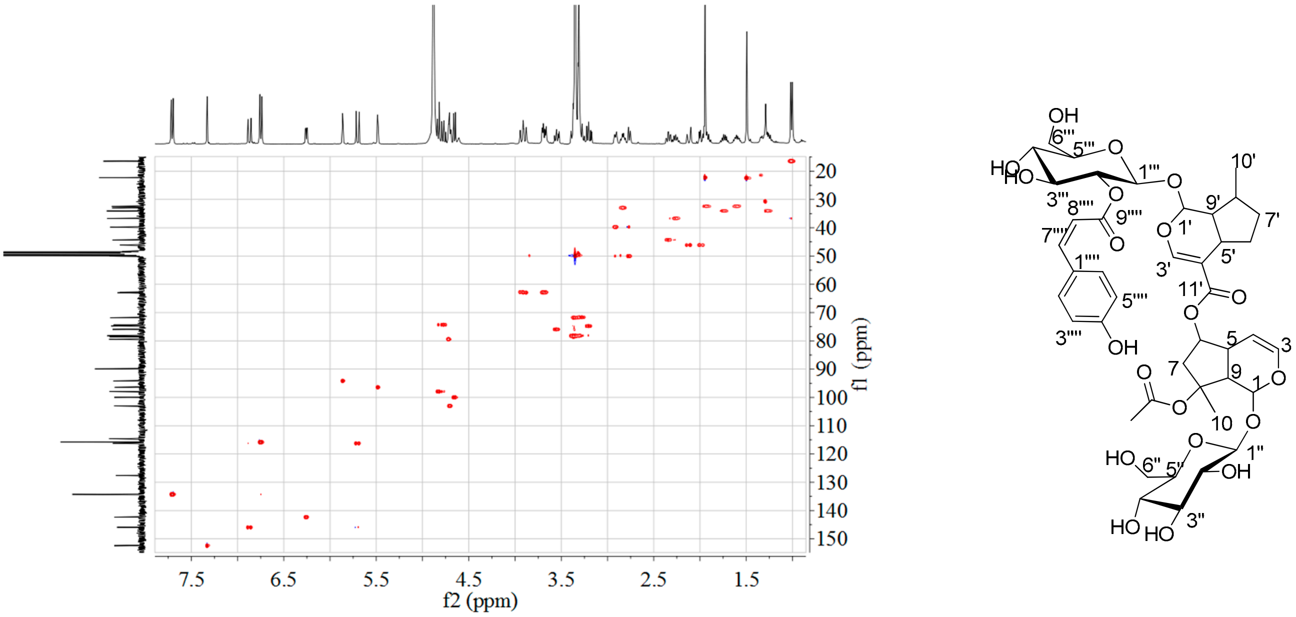

Figure S27. HSQC spectrum of 2‴-*O*-(*Z*)-*p*-coumaroyl caryocanoside B (**4**) in MeOD.

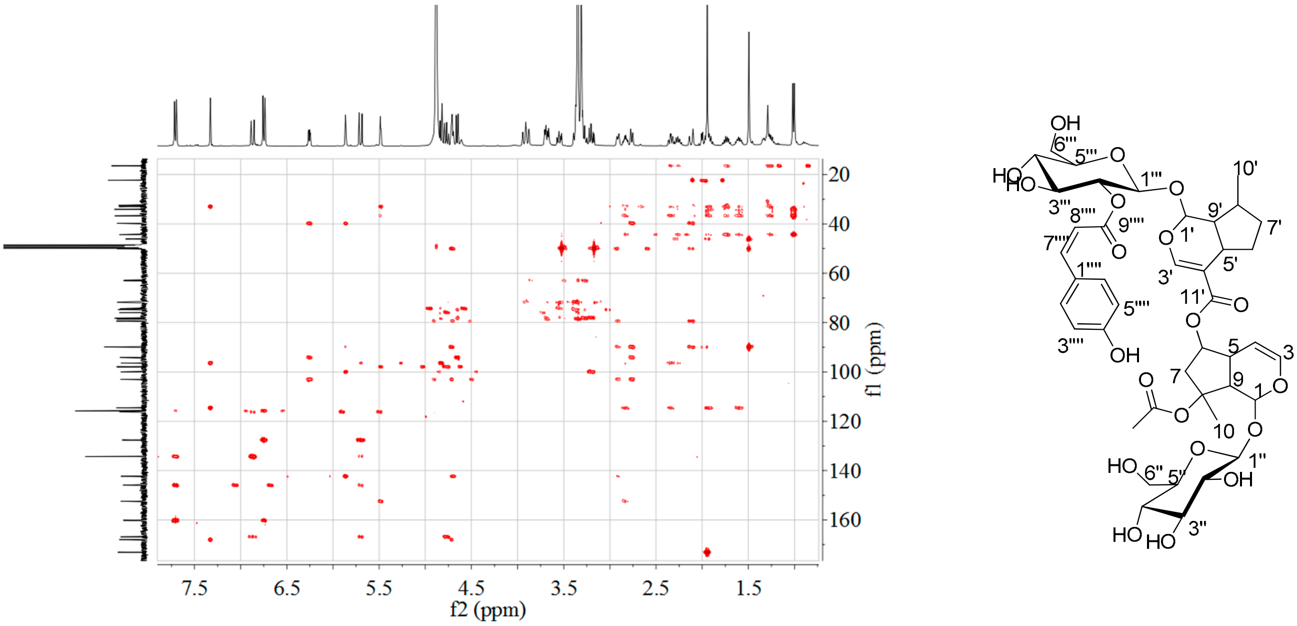

Figure S28. HMBC spectrum of 2‴-*O*-(*Z*)-*p*-coumaroyl caryocanoside B (**4**) in MeOD.

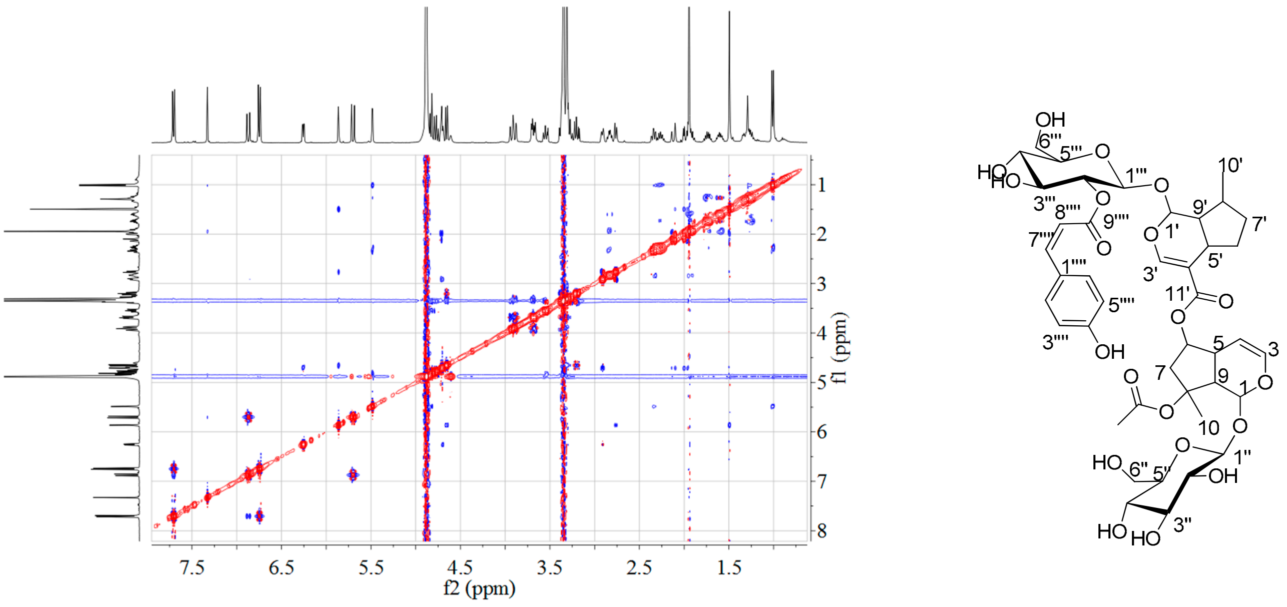

Figure S29. NOESY spectrum of 2‴-*O*-(*Z*)-*p*-coumaroyl caryocanoside B (**4**) in MeOD.

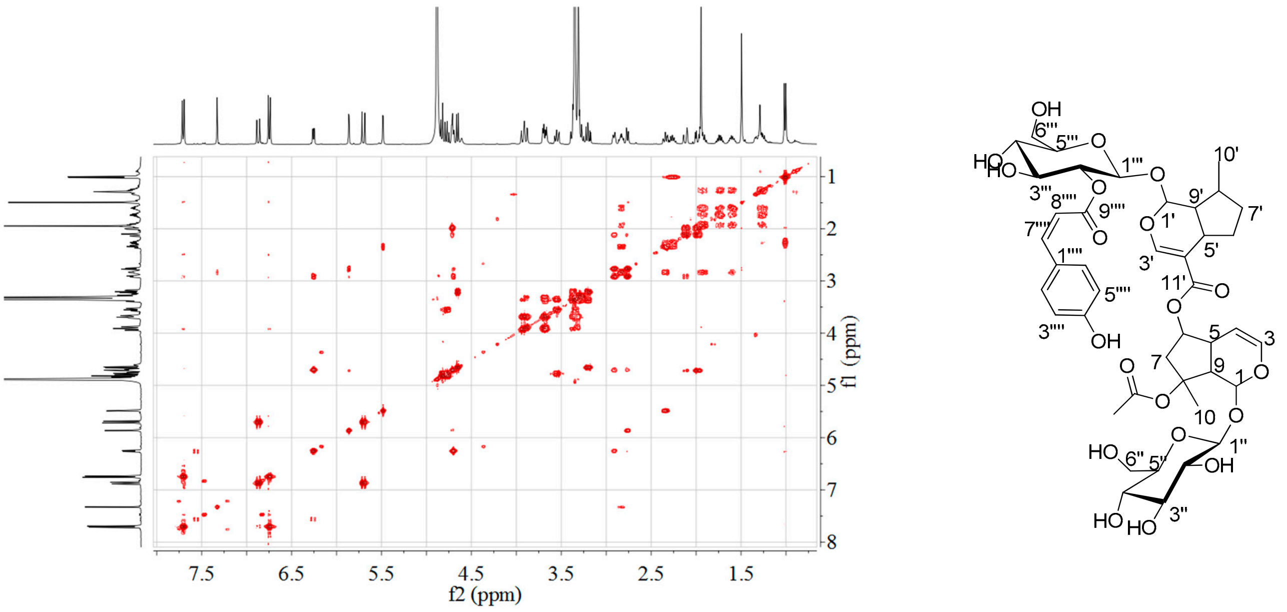

Figure S30. ^{1}H-^{1}H COSY spectrum of 2‴-*O*-(*Z*)-*p*-coumaroyl caryocanoside B (**4**) in MeOD.

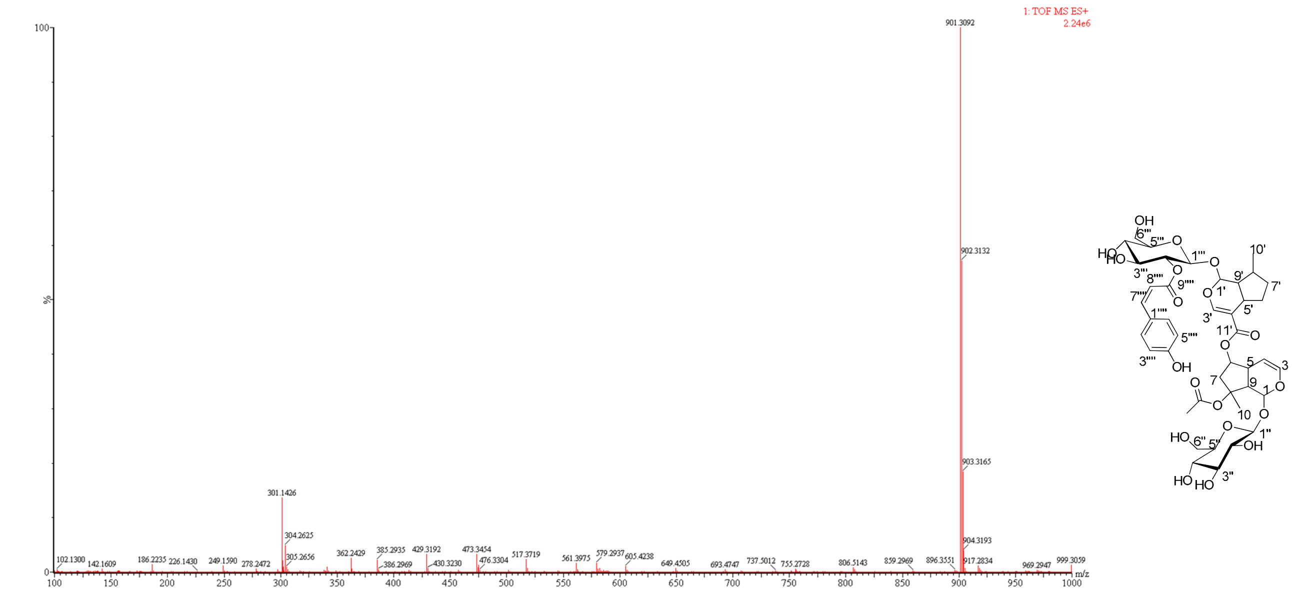

Figure S31. HRESIMS spectrum of 2‴-*O*-(*Z*)-*p*-coumaroyl caryocanoside B (**4**)

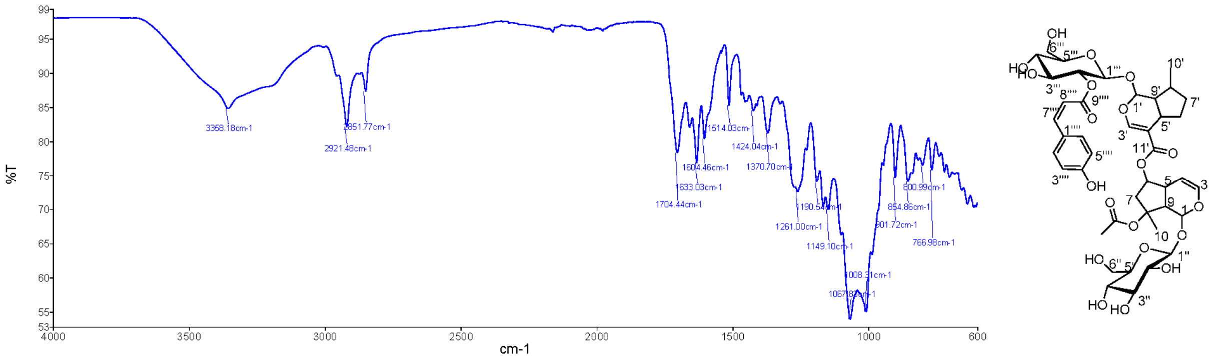

Figure S32. IR spectrum of 2‴-*O*-(*Z*)-*p*-coumaroyl caryocanoside B (**4**).

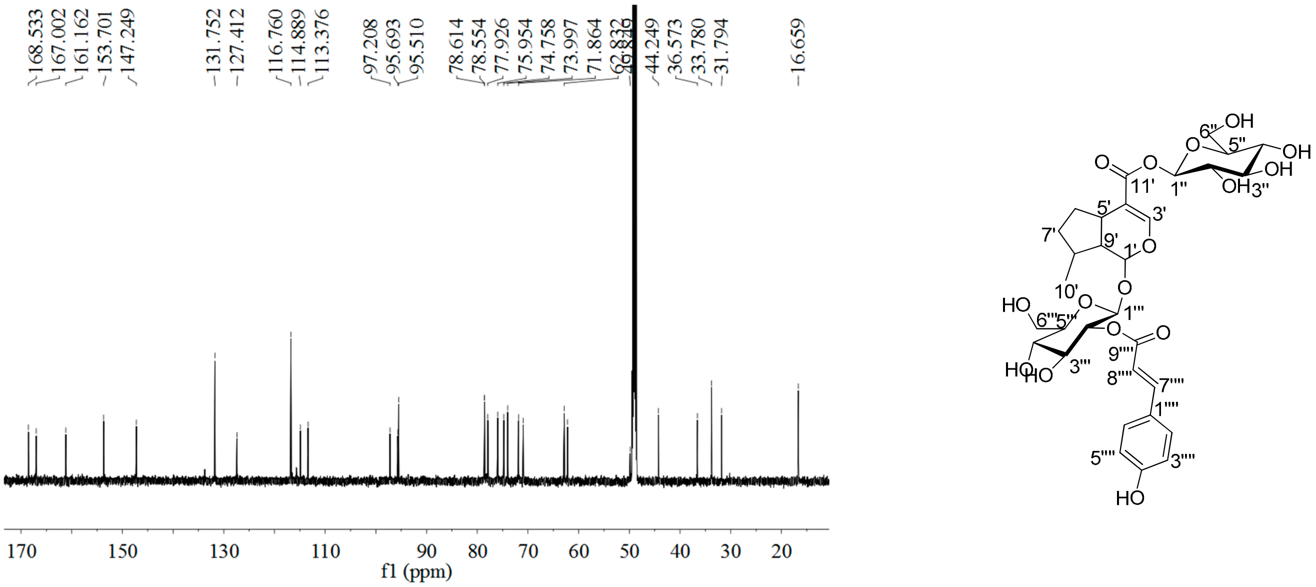

Figure S33. ^{13}C NMR spectrum of 2′-*O*-(*E*)-*p*-coumaroyl asystasioside A (**5**) in MeOD (150 MHz).

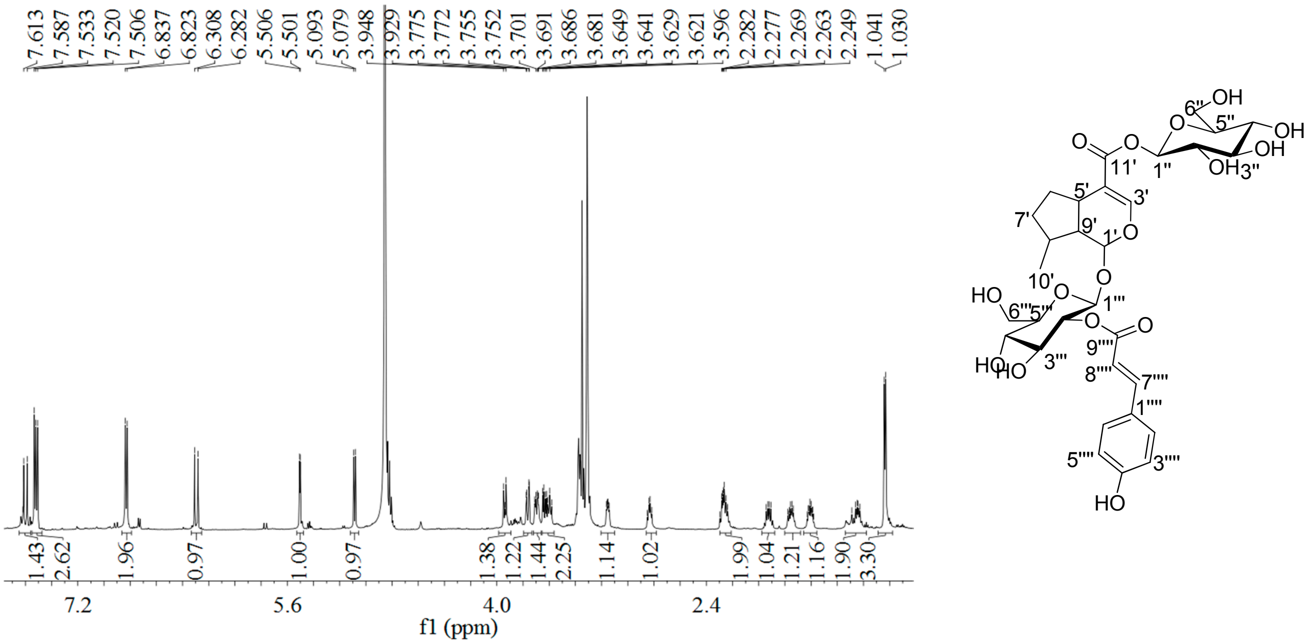

Figure S34. ^{1}H NMR spectrum of 2′-*O*-(*E*)-*p*-coumaroyl asystasioside A (**5**) in MeOD (600 MHz).

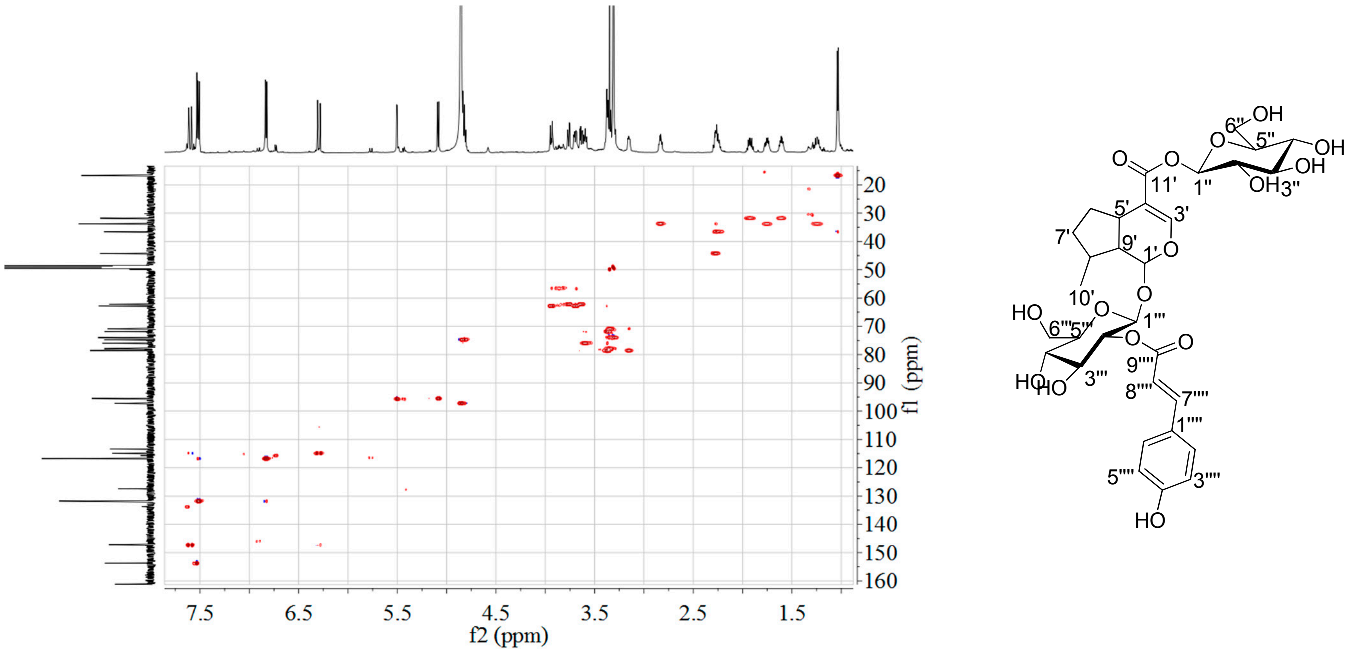

Figure S35. HSQC spectrum of 2′-*O*-(*E*)-*p*-coumaroyl asystasioside A (**5**) in MeOD.

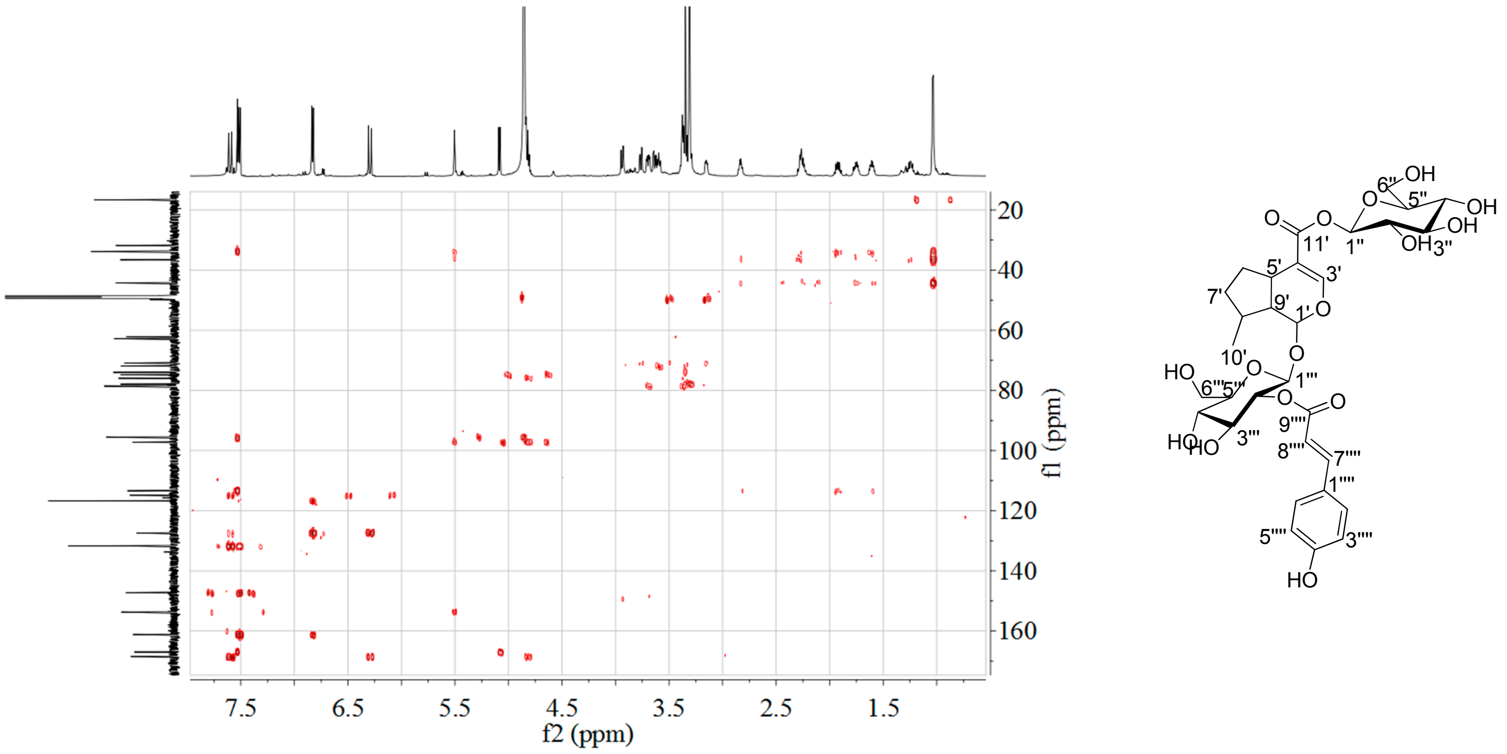

Figure S36. HMBC spectrum of 2′-*O*-(*E*)-*p*-coumaroyl asystasioside A (**5**) in MeOD.

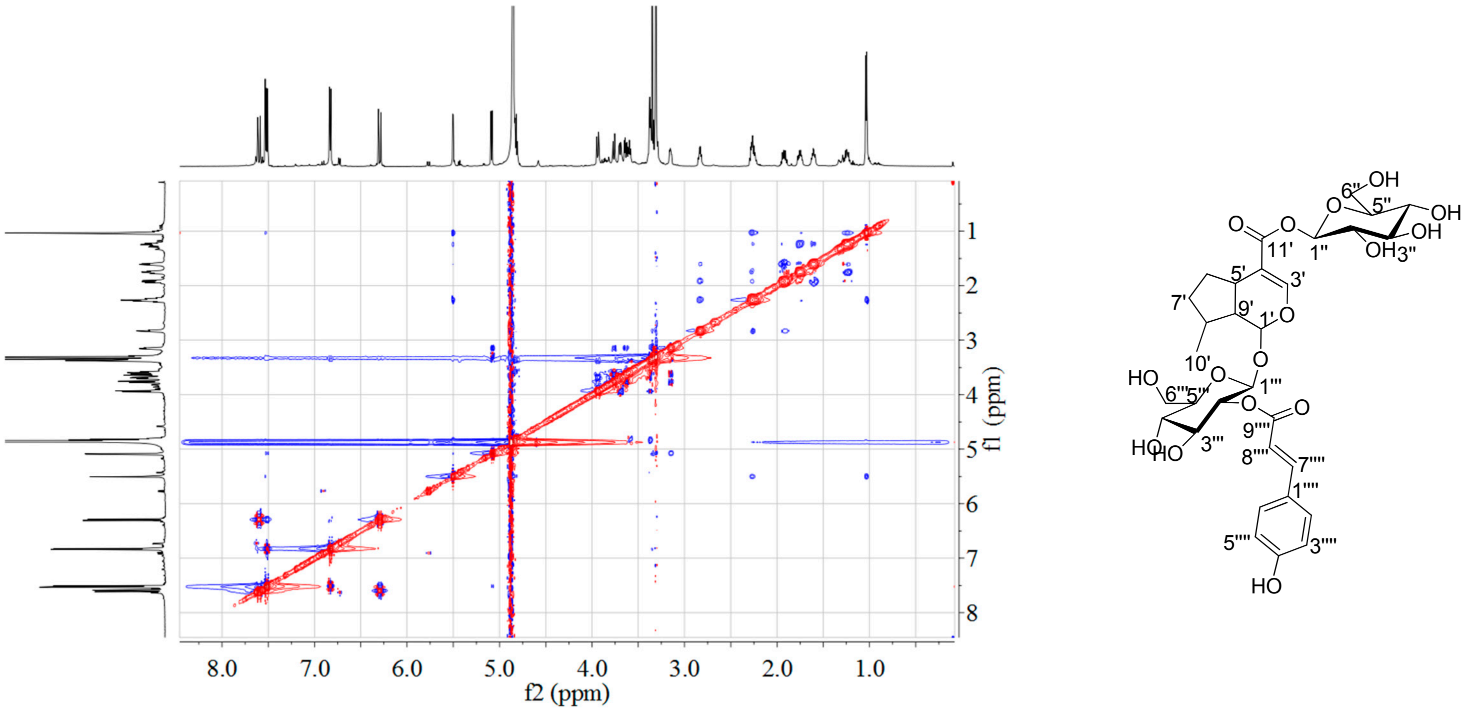

Figure S37. NOESY spectrum of 2′-*O*-(*E*)-*p*-coumaroyl asystasioside A (**5**) in MeOD.

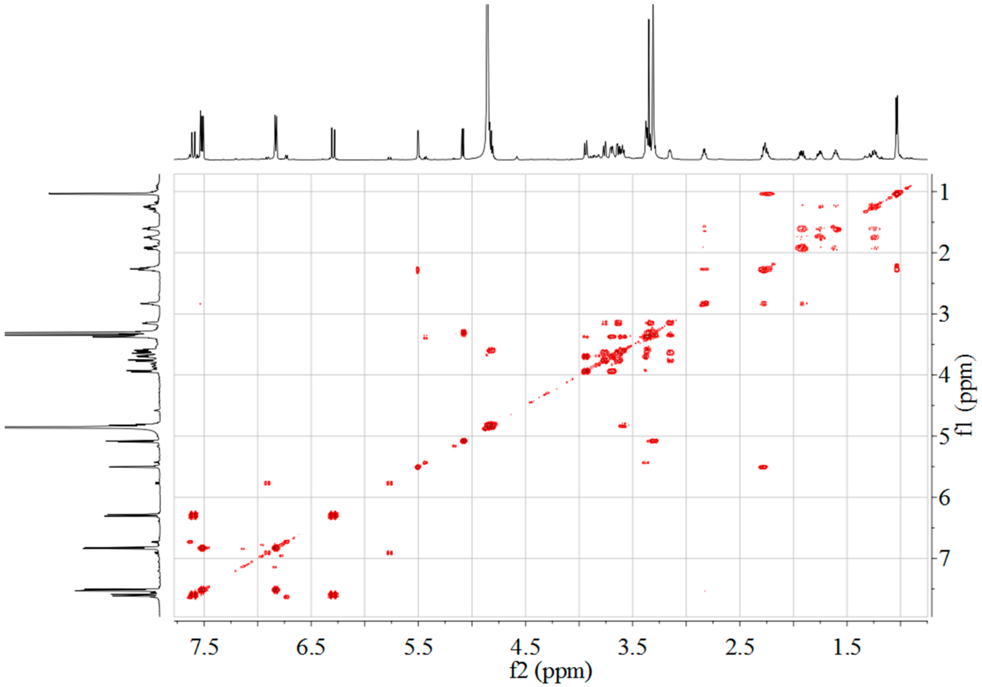

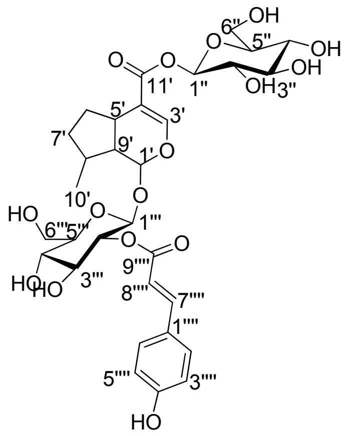

Figure S38. ^{1}H-^{1}H COSY spectrum of 2′-*O*-(*E*)-*p*-coumaroyl asystasioside A (**5**) in MeOD.

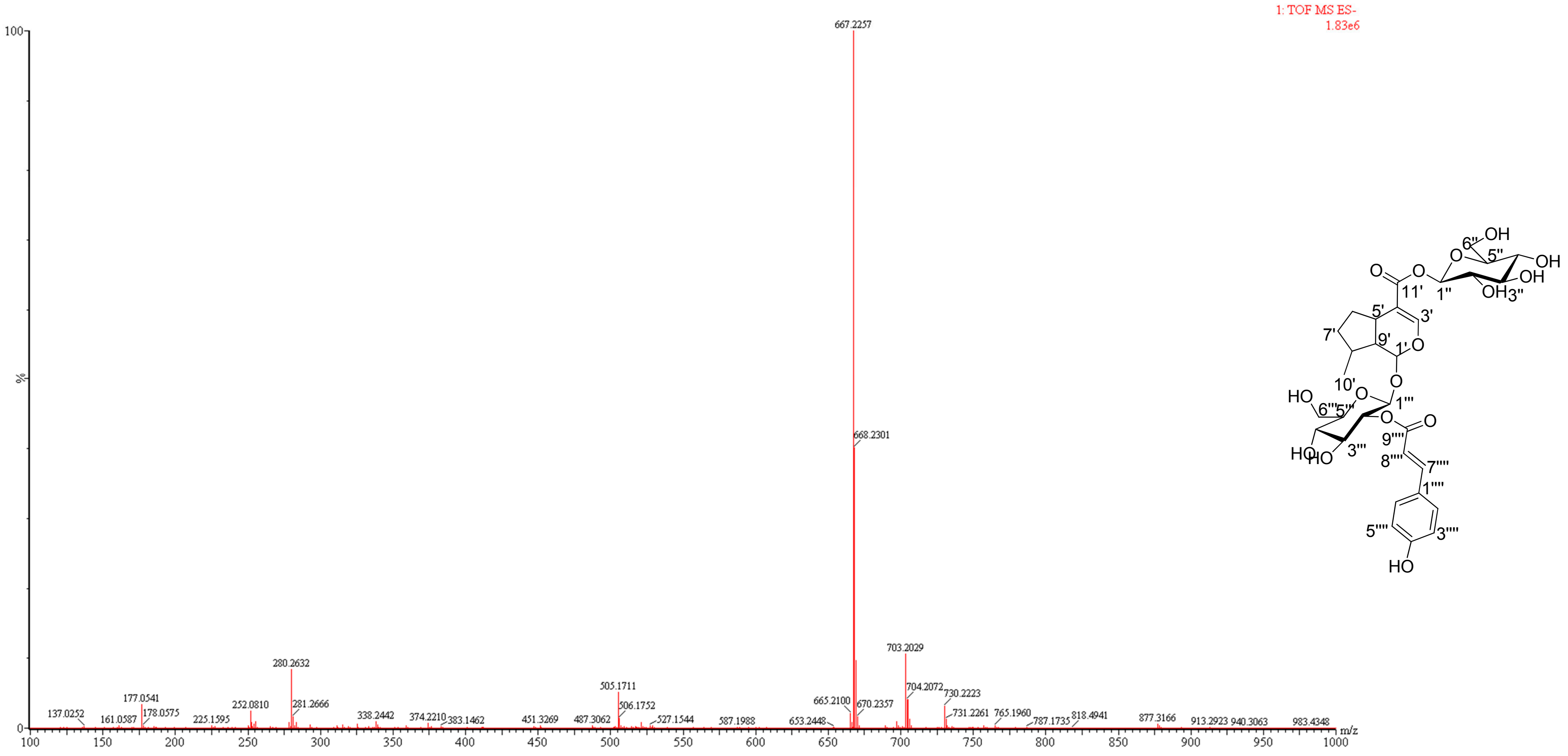

Figure S39. HRESIMS spectrum of 2′-*O*-(*E*)-*p*-coumaroyl asystasioside A (**5**).

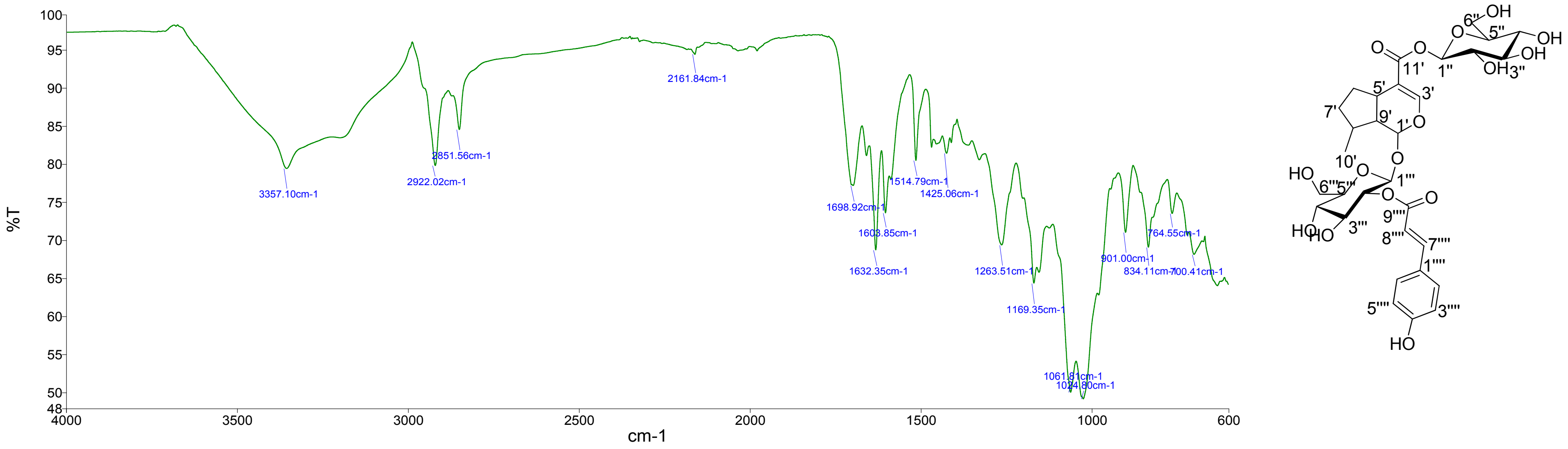

Figure S40. IR spectrum of 2′-*O*-(*E*)-*p*-coumaroyl asystasioside A (**5**).

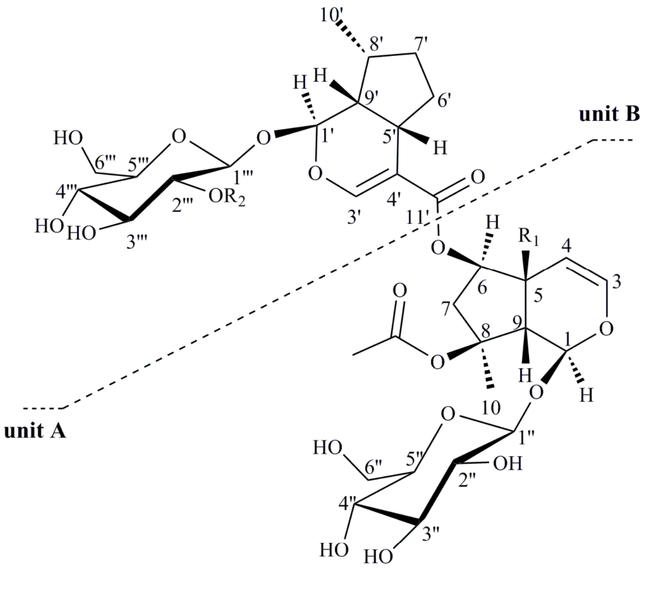

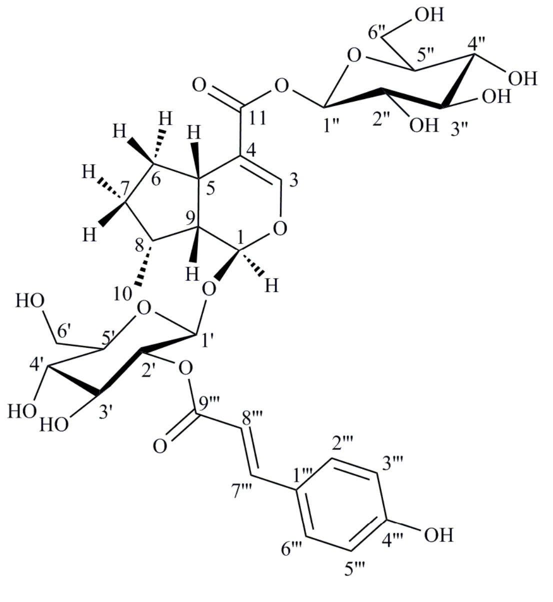

1 R_1 = H, R_2 = H
2 R_1 = OH, R_2 = caffeoyl
3 R_1 = H, R_2 = (*E*)-*p*-coumaroyl
4 R_1 = H, R_2 = (*Z*)-*p*-coumaroyl

5

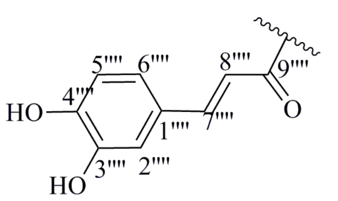

caffeoyl

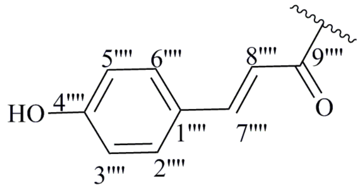

(*E*)-*p*-coumaroyl

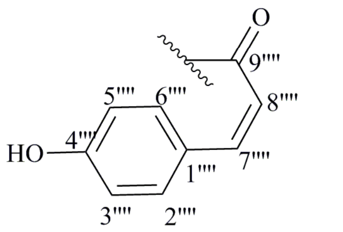

(*Z*)-*p*-coumaroyl

Figure S41. Chemical constitution of compounds **1**–**5**.

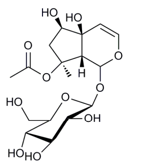

8-*O*-acetylharpagide (**6**)

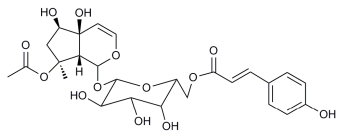

8-acetyl-6'-*O*-(*p*-coumaroyl)harpagide (**7**)

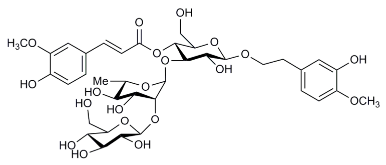

galactosylmartynoside (**8**)

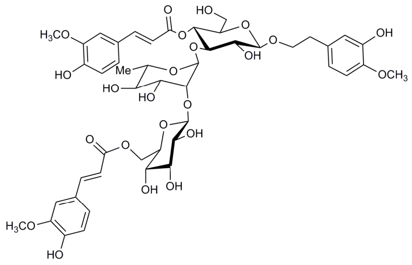

6'''-*O*-feruloylincanoside D (**9**)

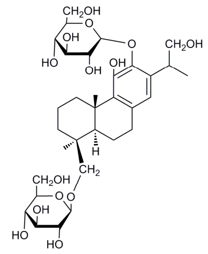

ajugaside A (**10**)

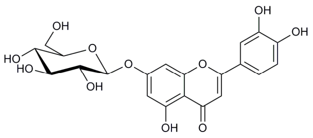

luteolin 7-*O*-glucoside (**11**)

Figure S42. Chemical constitution of compounds **6**–**11**.

Table S1. In vitro α-glucosidase inhibitory activity of compounds **1**–**11**.

Compounds	IC_{50}	Compounds	IC_{50}
1	>5.0	7	1.38 ± 0.27
2	>5.0	8	0.82 ± 0.15
3	>5.0	9	>5.0
4	0.38 ± 0.015	10	0.33 ± 0.06
5	3.35 ± 0.12	11	>5.0
6	1.89 ± 0.7	acarbose	3.49 ± 0.15
